# The PCOS–NAFLD Multidisease Phenotype Occurred in Medaka Fish Four Generations after the Removal of Bisphenol A Exposure

**DOI:** 10.1021/acs.est.3c01922

**Published:** 2023-08-15

**Authors:** Sourav Chakraborty, Santosh Anand, Seraiah Coe, Beh Reh, Ramji Kumar Bhandari

**Affiliations:** Department of Biology, University of North Carolina at Greensboro, Greensboro 27412 North Carolina, United States

**Keywords:** bisphenol A, NAFLD, PCOS, medaka, transcriptome, metabolome, transgenerational inheritance

## Abstract

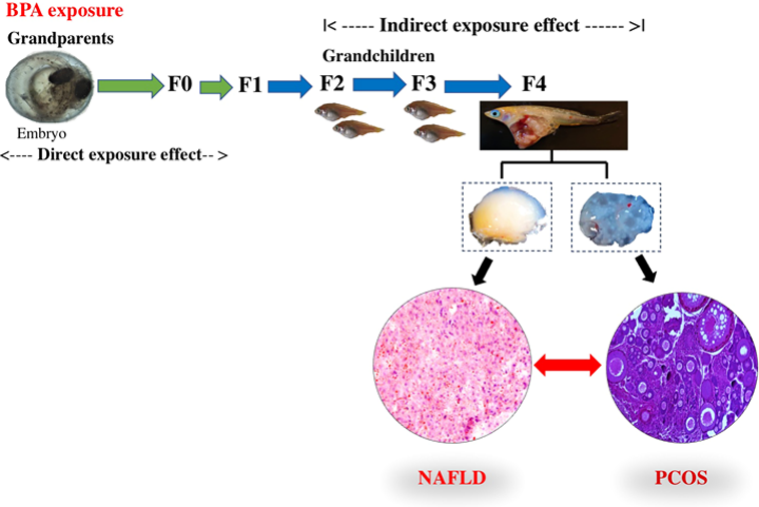

As a heterogeneous reproductive disorder, polycystic ovary syndrome (PCOS) can be caused by genetic, diet, and environmental factors. Bisphenol A (BPA) can induce PCOS and nonalcoholic fatty liver disease (NAFLD) due to direct exposure; however, whether these phenotypes persist in future unexposed generations is not currently understood. In a previous study, we observed that transgenerational NAFLD persisted in female medaka for five generations (F4) after exposure to an environmentally relevant concentration (10 μg/L) of BPA. Here, we demonstrate PCOS in the same F4 generation female medaka that developed NAFLD. The ovaries contained immature follicles, restricted follicular progression, and degenerated follicles, which are characteristics of PCOS. Untargeted metabolomic analysis revealed 17 biomarkers in the ovary of BPA lineage fish, whereas transcriptomic analysis revealed 292 genes abnormally expressed, which were similar to human patients with PCOS. Metabolomic–transcriptomic joint pathway analysis revealed activation of the cancerous pathway, arginine–proline metabolism, insulin signaling, AMPK, and HOTAIR regulatory pathways, as well as upstream regulators *esr1* and *tgf* signaling in the ovary. The present results suggest that ancestral BPA exposure can lead to PCOS phenotypes in the subsequent unexposed generations and warrant further investigations into potential health risks in future generations caused by initial exposure to EDCs.

## Abstract 2

Polycystic ovary syndrome (PCOS) can develop in females several generations after withdrawing from BPA exposure, indicating the possibility for past exposure effects persisting in future generations even after environmental remediation.

## 1 Introduction

Since the middle of the 20th century, there has been substantial growth in the development and production of industrial chemicals. BPA is one of the most widely manufactured chemicals and an environmental contaminant worldwide. BPA can induce epigenetic alteration in germline stem cells and gametes.^1−3^ Aside from causing adverse reproductive and nonreproductive health outcomes in the directly exposed generations, BPA can also contribute to transgenerational health outcomes in several subsequent generations.^4,5^ Various BPA-induced transgenerational health effects have been reported in animal models, such as diminished fertilization rate^6,7^ and osmoregulatory gene expression,^8^ cardiac disorders,^9,10^ social recognition and behavioral variations,^11^ and reproductive and metabolic diseases.^5,12^ Because of its widespread health effects, BPA has been banned in North America and the European Union in certain consumer goods.^13−15^ Although BPA is currently banned, the current ban may not completely protect future generations’ reproductive and metabolic health because ancestral BPA exposure-induced epigenetic changes in germ cells can be passed on to somatic cells of future generations, leading to impaired metabolic and reproductive health.^4,5^ We have previously demonstrated that ancestral BPA exposure could cause transgenerational nonalcoholic fatty liver disease (NAFLD), which can persist for five generations in medaka.^16^ In fish, the liver plays an intrinsic role in female reproduction by providing the major egg yolk precursor protein, i.e., vitellogenin, that supports embryo development and larvae by supplementing protein and lipid-rich nutrients.

Typically, ovarian function depends largely on the hypothalamus–pituitary–ovary (HPO) axis activated by GnRH pulsatility followed by releases of pituitary-derived gonadotropins such as follicle-stimulating hormone (FSH), luteinizing hormone (LH), and sex steroids (estrogen, progesterone, testosterone) secreted by gonads.^17,18^ As the most common female reproductive disease, polycystic ovarian syndrome (PCOS) is characterized by an imbalance between FSH and LH, inhibited follicle maturation, formation of multiple small cysts due to degeneration of follicles, and an increase in free testosterone.^19,20^ Up to 75% of women of reproductive age suffer from PCOS due to hormonal imbalance, making it the most prevalent endocrine disease among premenopausal women.^21^ As an acute endocrine disruptor, BPA dysregulates FSH and LH levels followed by abnormal folliculogenesis,^22^ hinders embryo implantation,^23^ alters estrous cyclicity,^24^ and upregulates AMH, causing an increase in the number of preovulatory follicles.^25^ BPA is presumed to be an epigenetically toxic compound.^26^ Aside from causing adverse health outcomes in the exposed generation, BPA can also contribute to transgenerational health outcomes through abnormal epigenetic changes in germ cells via germline transmission.^27,28^ In a previous study, polycystic ovarian syndrome was positively correlated with metabolic diseases such as NAFLD^29^ and deregulation of estrogen signaling,^30−32^ suggesting cross talk between the hepato-ovarian axis. A positive correlation has been found between PCOS and biopsy-confirmed NAFLD^33−35^ at an intragenerational level; however, whether this relationship is heritable at the transgenerational level is not currently understood. The proposed study further investigates this issue by incorporating phenotypic and omics approaches.

A multidisease phenotype can develop in organisms due to direct exposure to environmental chemicals; however, the mechanisms underlying such compounded adverse health outcomes are not clearly understood. Neither is it clearly understood whether such phenotypes persist in organisms in subsequent generations after the remediation of the environmental contamination. Using a multi-omics (integrated transcriptomics and metabolomics) approach, the present study examined heritable NAFLD–PCOS, a multidisease phenotype, in medaka fish whose ancestors were exposed to BPA during their first 15 days of life and never thereafter. Fish, such as medaka and zebrafish, serve as biomedical animal models to study human diseases, including NAFLD and PCOS.^36−40^ Because of the conserved mechanism for processing epigenetic information in post-fertilization stage embryos and primordial germ cells with mice and humans, medaka becomes an ideal animal model to study the environmentally induced epigenetic inheritance of transgenerational disease phenotype.^39,41^

## 2 Materials and Methods

### 2.1 Animal Care and Maintenance

The Hd-rR strain of medaka was used.^42^ The exposure and procedure for handling fish and euthanization were approved by the Institutional Animal Care and Use Committee (IACUC) of the University of North Carolina Greensboro (#20-002). Adult medaka fish were maintained in 20 L glass aquaria on a light–dark cycle of 14:10 h with a recirculatory water system with an exchange of 25% water every 4 h at 26 ± 1 °C and fed three times a day with Otohime granular food and newly hatched brine shrimp (*Artemia* nauplii). Embryos were incubated in glass Petri dishes and larvae in 1 L tanks until they could feed and swim independently. The larvae at above 25 dpf stage were transferred to 5 gal glass tanks for rearing until end point measurement. For this study, 4 month old adult medaka females were used.

### 2.2 Chemical Exposure

Bisphenol A exposure can affect metabolic and reproductive health at various concentrations.^43−47^ The present study is the downstream analytical part of a bigger transgenerational study. The concentration of BPA was selected after testing a dose–response curve, and the concentration of BPA selected was 10 μg/L, which is realistic with regard to the environmental concentration in many regions of the world.^48,49^ The measured concentration of BPA was within <10% of the calculated concentration throughout the exposure period, and uptake was measured to be 20 pg/mg egg/day as previously described.^48,49^ BPA exposure was initiated 8 h post fertilization stage (hpf) and continued until day 15 after fertilization (dpf) with a renewal of exposure once daily. BPA exposure was designed to include epigenetic reprogramming events in germ cells and to avoid epigenetic reprogramming of embryos that takes place between fertilization and blastula stages (8 hpf). After the BPA exposure was complete, the F0 generation (exposed individuals) and subsequent generations (offspring) were raised in clean water without exposure to BPA. The exposure window thus included a critical developmental window for sex determination and liver differentiation.^50^ The use of initial BPA exposure (10 μg/L) at F0 generation was previously reported to cause NAFLD, fertilization defects, and increased embryo mortality in subsequent unexposed generations of medaka.^8,16,48^

### 2.3 Production of BPA-Exposed Transgenerational Offspring

Generation of the BPA-exposed transgenerational lines of medaka was previously described.^51^ Briefly, following BPA exposure for 15 days, larvae were raised in clean water until they became adults, designated as F0 adults (intragenerational due to direct exposure to BPA). At 120 days postfertilization, all experimental medaka reached sexual maturity and spawned.^52^ A total of six pairs of fish from the F0 generation were bred at 120 days postfertilization to produce F1 (intergenerational). The same mating methodology was used to produce subsequent generations up to F4 (the fifth generation, transgenerational).

### 2.4 Fecundity and Fertilization Efficiency of the F4 Fish

The fish from BPA-exposed and control lineages were bred in glass tanks at a ratio of three females and two males for 7 days. Each morning, eggs laid by females were collected and examined under a microscope to determine fecundity and fertilization status. Fecundity was calculated as the average number of eggs laid per female per day. Eggs were examined under a stereoscope to confirm fertilization according to the methods previously described.^53^ A two-tailed *t* test was performed between the F4 generation of BPA lineage and the control group to determine statistical significance.

### 2.5 Tissue Sample Collection

To examine whether the BPA lineage fish develop PCOS-like syndrome in adulthood (120 dpf), 15 females from each group (BPA lineage and control lineage) were euthanized with MS-222 (250 mg/L). The ovary and liver of both groups were dissected, weighed, and divided into two halves. For RNA/DNA extraction, half of the ovary and liver were fixed in RNA/DNA shield solution (Zymo Research, CA) and the other half in Bouin’s solution for histopathological analysis. Hepatosomatic index (HSI), gonadosomatic index (GSI), and body mass index (BMI) were calculated as liver weight (g) × 100/body weight (g), ovary weight (g) × 100/body weight (g),^54^ and [body weight (g) – ovary weight (g)]/body length (cm^2^), respectively. Visceral fat was collected to quantify adipose tissue (see Supporting Information). For histological examination, samples were embedded in low-temperature paraffin, sectioned at 5 μm thickness in a microtome, and stained with hematoxylin following optimized histological protocol.^16^ To determine fat accumulation, liver samples from the control and BPA lineages were fixed in the Optimal Cutting Temperature (OCT) compound, sectioned in a cryotome at 15 μm thickness, and used for Oil Red O staining.

### 2.6 Ovary Phenotyping

On the basis of follicular development, the ovary of the medaka was categorized into five developmental stages according to the literature previously published.^55^ Stage I contained primary follicles with intense basophilic cytoplasm with a peripherally located nucleus. Stage II contained cortical alveolar stage oocytes. Stage III contained yolk in vitellogenic oocytes with conspicuous zona radiata. Stage IV contained abundant yolk granules in late vitellogenic oocytes with peripheral migration of the germinal vesicle. Stage V contained mature stage oocytes without germinal vesicles, according to the published literature.^56^ Developmental stages of the ovary were measured in both groups (BPA lineage and control), and the two-way analysis of variance was used to evaluate the significance of differences in different stages of folliculogenesis.

Hyperplasia of granulosa cells; thinning, invasion, and breakdown of the zona radiata; disintegration of the basal membrane; and absorption of vitellus are the characteristics of atretic follicles.^57^ The ImageJ software was used to measure the follicular area. The atretic follicles from control and BPA-lineage fish were analyzed by measuring the follicular area using the ImageJ software. The reference value was taken from the mean area of the atretic follicles (1p^2^) in the control ovaries. Atretic follicles with a diameter greater than 1p^2^ were considered as big atretic follicles. Blind tests were conducted by two different lab members to characterize different stages of folliculogenesis and atretic follicles. The results were consistent among all testers.

### 2.7 RNA-Seq

#### 2.7.1 Library Preparation, RNA Sequencing, and Data Analysis

The ovaries of control and BPA lineage female fish (15 individuals from each lineage) were used for total RNA extraction by using a Quick RNA/DNA Miniprep Plus Kit (#D-7003, Zymo Research, CA, USA) according to the manufacturer’s protocol as previously described.^58^ RNA quality was tested by bleach gel electrophoresis,^59^ and the quantity was determined by Nanodrop 2000 and Qubit (Thermo Fisher, Waltham, MA). The RNA of the ovary from three females was pooled as one biological replicate per group. Transcriptome libraries were prepared using a NEBNext Ultra II RNA Kit and the manufacturer’s protocol. The libraries were sequenced on Illumina HiSeq X (Novogene Corporation, CA, USA) using a 150 bp paired-end sequencing strategy (short reads), producing 20–40 million reads per biological replicate.

The reads were first preprocessed with fastp 0.23.2,^60^ an ultrafast all-in-one FASTQ preprocessor, which performs quality control, adapter trimming, quality filtering, per-read quality pruning, and many other operations with a single scan of the FASTQ data. The processed reads were then mapped to the medaka genome (Oryzias_latipes.ASM223467v1) using STAR 2.7.7a.^61^ Finally, DESeq2 v1.34.0 was used to do the differential expression analysis.^62^ GSEA analysis was done using the GSEA Preranked tool (GSEA v4.1).^63,64^ For GSEA analysis, only genes showing twofold up- or downregulation were considered. The Gene Ontology (GO) enrichments were obtained using “C5: ontology gene sets” of the MSigDB collection.^65^

#### 2.7.2 Comparative Analysis of the BPA Lineage Ovary Gene Set with the Human PCOS Patient Data Set

A predefined set of PCOS specific DEGs was obtained from public human patient data sets: GSE34526125, GSE10946126, and ArrayExpress accession number E-MEXP-3641127.^66−68^ The DEGs (FDR 0.05 and Log2FC > 0.5) from the medaka ovary RNA-seq were compared with the PCOS patient data set.^66^ Overlapping DEGs between BPA lineage and PCOS patient group were selected and illustrated by using VENNY (http://bioinfogp.cnb.csic.es/tools/venny/index.html). The PCOS-specific DEGs represented in the ovary of the BPA lineage group were used, and predicted pathways were constructed using ShinyGO 0.76.3.^69^

### 2.8 RT-qPCR

Total RNA was isolated from the ovary and liver of the F4 generation fish using a Quick RNA/DNA Miniprep Plus Kit (#D-7003, Zymo Research, CA, USA) according to the manufacturer’s protocol involving *Dnase I* treatment of RNA as previously described.^58^ The RNA was reconstituted in 20 μL of nuclease-free water (Zymo Research, CA, USA) followed by testing RNA integrity by bleach gel electrophoresis^59^ and quantified by Qubit and Nanodrop 2000 (A260/280 between 1.8 and 2). Reverse transcription of 1 μg of total RNA was performed on each sample using a high-capacity reverse transcription kit (Applied Biosciences) according to the manufacturer’s instructions as previously described.^53,70^ RT-qPCR was performed in a QuantStudio 3 Real-time PCR equipment (Applied Biosystems) using gene-specific primers (Table S1) and the 2^–ΔΔCt^ method. The *rpl7* gene was selected as a stable housekeeping gene after testing the expression pattern of several housekeeping genes. Primers were designed using Primer3web^71^ from exon–exon junctions to avoid genomic DNA amplification (Table S1).

### 2.9 Ingenuity Pathway Analysis (IPA) of DEGs

Canonical pathways were determined by using the IPA software (V01-07; Qiagen), and molecular and cellular function were determined using the specific Ingenuity Knowledge Database (using default parameters for all tissues and cell lines, with relaxed filters), which provides a repository of biological interactions and functional annotations. Dysregulated genes (FDR 0.05 and Log2FC > 0.5) in the ovary of F4 generation fish were converted to the corresponding human orthologous using the g-profiler website.^72^ Fisher’s exact test was applied to calculate the significance of each network.

### 2.10 Metabolomic Quantification

The ovaries of F4 females from control and BPA lineage were pooled respectively and used to extract ovarian metabolites. According to a published protocol,^73^ ovary samples (20 mg) were homogenized in an ultrasonically extracted solution consisting of methanol/acetonitrile/water (2:2:1, v/v), vortex mixed, and ultrasonically extracted twice at a low temperature for 30 min. After incubation at −20 °C for 1 h, the mixture was centrifuged at 13,000 rpm at 4 °C for 15 min. The supernatant was lyophilized and stored at −80 °C. Before use, the lyophilized sample was dissolved in acetonitrile water (1:1, v/v), vortex-mixed, and centrifuged at 14,000 rpm for 15 min. The supernatant was injected into the HPLC–MS/MS system for metabolomic analysis. To ensure the system’s stability, eight quality control (QC) samples, including whole adductor muscle tissues, were inserted throughout the experiment. Mass spectrometry was performed on a Q Exactive plus (Thermo Fisher Scientific, Waltham, MA, USA) equipped with electrospray ionization. The mass spectrometry operational protocol was previously published.^74^ Both negative and positive ion modes were applied with a capillary voltage setting of ±5.5 kV during instrument operations. The product ion scan *m*/*z* range was 25–1200 Da with a scan accumulation time of 0.03 s/spectrum. The collision energy was set to 30 eV.

### 2.11 Metabolomic Data Analysis

Mass spectrometric data were analyzed, aligned, and filtered with the MZmine 2.2 software (http://mzmine.sourceforge.net/) using the procedure previously reported elsewhere.^74^ Quantile normalization, cube root transformation, and mean centering were used to process the filtered peaks from 4232 peaks in the positive mode and 3569 peaks in the negative mode. The resulting features were used to explore the total pathway hit by the Mummichog algorithm installed in Metaboanalyst 5.0.^75^ Metabolites associated with pathways were further used for PCA to reveal intrinsic feature clusters and detect outliers. To examine the relationship between group and spectral data with variance (R2Y) and predictive ability (Q2 parameter), a PLS-DA model was used. The resonance of metabolites was putatively annotated bioinformatically by using the HMDB database^76^ (http://www.hmdb.ca) and metlin^77^ (http://metlin.scripps.edu). To visualize the variations of metabolites between the control and BPA lineage group, the significantly altered metabolites in the BPA lineage compared with the control lineage were identified based on the following criteria: *P* < 0.05 and fold change ≥ ±1. VIP > 1 was used for hierarchical clustering (Ward clustering, Euclidean distance), univariate analysis *t* test, and pathway analysis using Metaboanalyst 5.0 as previously described.^78^ To determine metabolomic biomarkers of PCOS, the classical univariate ROC curve analysis was performed.^79^ The area under the curve (AUC) was generally between 0.5 and 1.0. When AUC > 0.5 and closer to 1, the model performs better.^80^ However, biomarkers were selected based on strict criteria: *P* < 0.05 and AUC = 1 from the ovary of the BPA lineage. BPA lineage ovary metabolites were compared with the PCOS patient metabolite data set.^81^ The abundance of amino acids in the BPA lineage ovary was plotted in metabolic pathways associated with polycystic ovary syndrome (PCOS).^82^

### 2.12 Statistical Analysis

The GraphPad Prism (GraphPad Software, San Diego, CA) software was used for statistical analysis and plotting the results. Comparison of data between BPA lineage and control lineage, Student’s *t* test, one-way analysis of variance (ANOVA), and post hoc multiple comparison tests (Tukey HSD) were used to determine significant differences. Differences with *P* < 0.05 were considered significant and have been marked with asterisks (*P < 0.05; **P < 0.01; ***P < 0.001).

## 3 Results

### 3.1 F4 Generation Female Fish from the BPA-Exposed Lineage Showed Altered Morphological End Points

**Figure 1:**
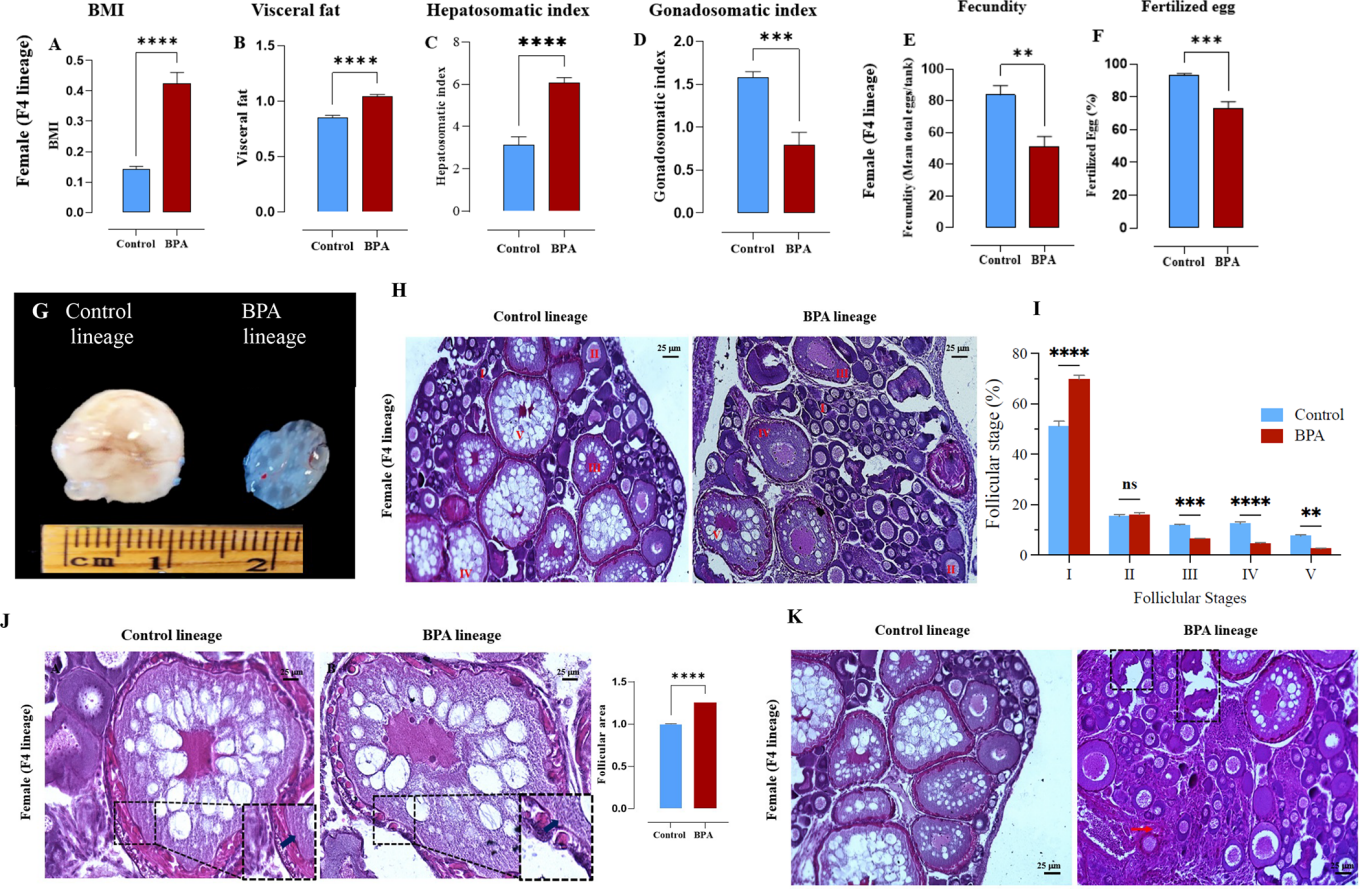
Ancestral BPA exposure caused increased basal metabolic index (BMI), visceral fat accumulation, and hepatosomatic index and reduced gonadosomatic index in BPA lineage females. (A) BMI (mass/length^2^), (B) visceral fat, (C) hepatosomatic index, and (D) gonadosomatic index. Statistical significance (***P < 0.001, *t* test) compared to control lineage. Ancestral BPA exposure perturbed maturation of ovarian follicles with fertilization defect. (E) Fecundity, (F) fertilized egg, and (G) size of the ovary. (H) Ovary histology (magnification ×40) micrograph in control and BPA lineage. (I) Number of follicles in each developmental stage in the ovaries of the BPA and control lineage fish. Asterisks indicate statistically significant differences (***P < 0.001, *t* test). I: primary growth stage; II: cortical alveolar stage; III: early vitellogenic stage; IV: late vitellogenic stage; V: mature stage; A: atretic follicle. (J) Degenerated follicle (black rectangle) with excess interstitial tissue deposition (red arrow) found in the ovaries of BPA lineage fish. (K) Atretic follicle with damaged chorion found in the ovary of BPA lineage (magnification ×40) and follicular area.

Body mass index (BMI) and gonadosomatic index (GSI) were measured to determine the metabolic health of the F4 females. Compared to the control lineage, the BPA lineage females had significantly increased BMI, visceral fat content (Figure 1A,B), and hepatosomatic index (Figure 1C). However, the GSI (Figure 1D) was significantly decreased, suggesting that the F4 fish from the BPA lineage had a reproductive impairment.

### 3.2 Ancestral BPA Led to Altered Reproductive Outcomes in Females

To determine whether the females had reproductive defects, fecundity was measured, which accounts for the production of eggs per female in the BPA and control lineage. The mean total production of eggs per tank (fecundity) and the number of fertilized eggs were significantly decreased (*P* < 0.05) in the BPA lineage group compared to the control group (Figure1E,F). A 40% decrease in ovary size was observed in the BPA lineage fish (Figure 1G) compared to the control lineage. The number of vitellogenic and postvitellogenic follicles was significantly decreased in the BPA lineage (Figure 4A,B) except for stage II (Figure 1H). Overall, a 90% decrease in the number of vitellogenic and postvitellogenic follicles was observed. Morphometric analysis of the follicular stages showed a significantly higher number of stage I oocytes in the ovary of the BPA lineage fish, which decreased across stages III through stage V (Figure 1J). This indicated that follicular progression was disrupted in the BPA lineage fish. Furthermore, the BPA lineage group showed damage in the chorion (Figure 1J) and an increased mean area of atretic follicles (3.067 ± 0.6190 p^2^, Figure 1J). Additionally, increased numbers of the atretic follicles, irregular folding of the surface epithelium, degenerated follicles with abnormal tissue deposition (Figure 1K), and hyperplasia of the granulosa cells were found in the ovary, indicating a loss of follicular architecture in BPA lineage fish.

### 3.3 Global Transcriptomic Alterations and GO Analysis in the Ovary

**Figure 2:**
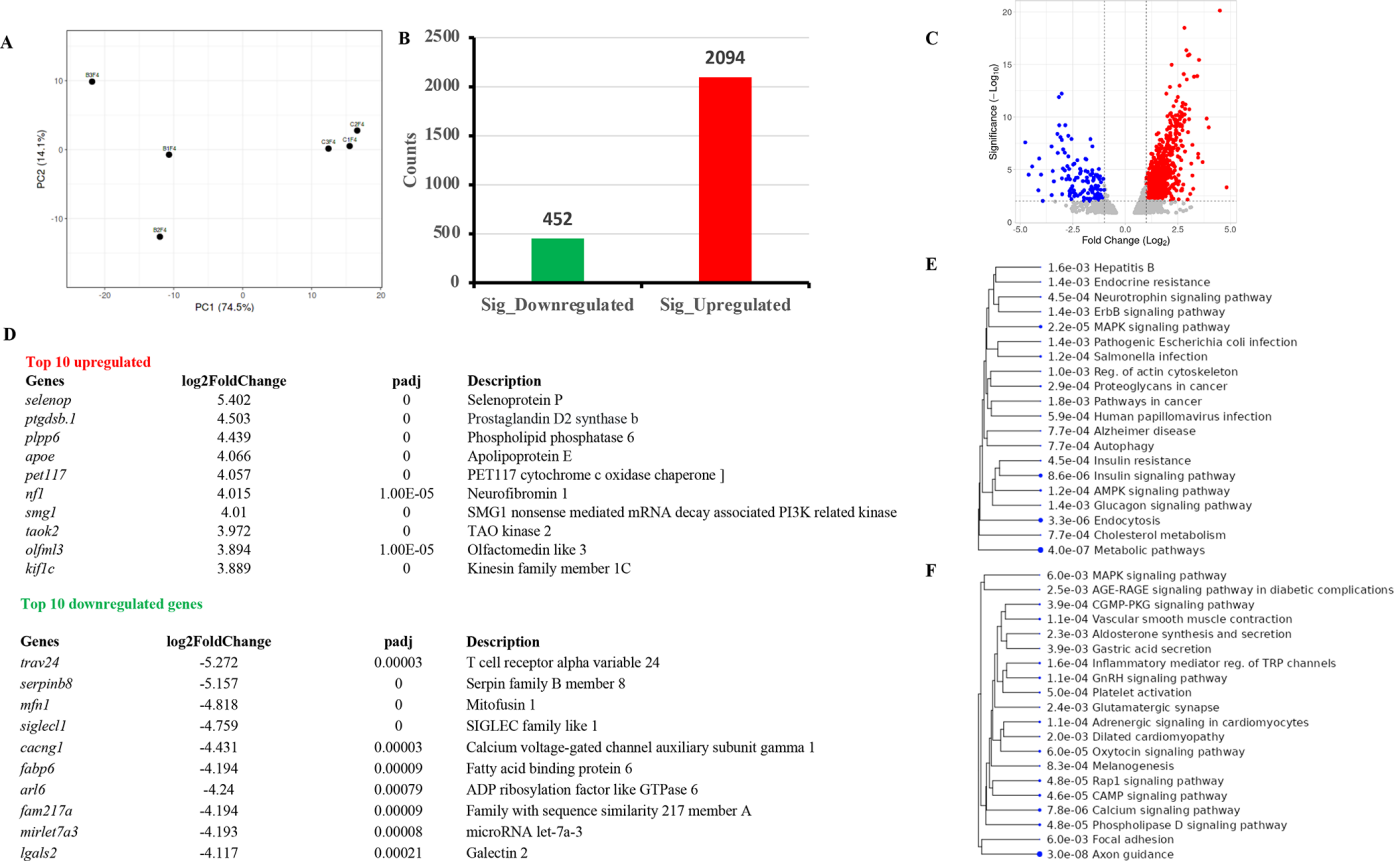
Global transcriptional alterations in the ovary. (A) PCA plot showing a separation of DEGs in BPA from control lineage ovaries. (B) Bar plots showing the profile of up- and downregulated genes and (C) log2 fold-change volcano plot. (D) Top 10 up- and downregulated genes in the ovary of BPA lineage. KEGG pathway analysis of total upregulated (E) and downregulated (F) genes in the transcriptome dataset.

Principal component analysis (PCA) was able to separate 74.5% of biological replicates of both the BPA lineage and control lineage group into two clusters (Figure 2A), indicating that they are different from each other. The expression profiles were consistent across all three biological replicates, indicating that sequencing libraries were of excellent quality. In total, 2546 differentially expressed genes (DEGs), including 2094 upregulated and 452 downregulated genes, were identified in the BPA lineage group (Figure 2B), as exhibited in the volcano plot (Figure 2C). Global alterations in gene expression in BPA lineage ovary are illustrated in a heatmap (Figure S1). The GO analysis was performed to evaluate the functional properties of DEGs. GO results showed the top 20 molecular functions, biological processes, and cellular components (Figure S2). Among all biological processes, cellular morphogenesis, differentiation, and protein localization were highly enriched by DEGs from the BPA lineage. Cellular components and molecular function had DEGs involved in microtubule organization, cellular adhesion, and cellular transport mechanism via the Golgi apparatus. Additionally, using stricter selection criteria (fold change ≥ 2, *p*-adj ≤ 0.01), the top 10 up- and downregulated DEGs (biomarker) were selected from the BPA lineage group (Figure 2D). Most upregulated genes were enriched in insulin signaling pathway (Figure 2E, Figure S3), MAPK signaling (Figure 2E, Figure S4), AMPK signaling pathway (Figure 2E, Figure S5), and autophagy pathway (Figure 2E). Alternatively, downregulated genes were enriched in Rap1 (Figure 2F, Figure S6), cAMP (Figure 2F, Figure S7), and AGE-RAGE signaling pathway (Figure 2F, Figure S7).

#### 3.3.1 Gene Set Enrichment Analysis (GSEA) of Ovary Transcriptome

GSEA found a positive correlation of upregulated genes with autophagic mechanism (Figure S8A), cellular stress response (Figure S8B), and chromatin organization (Figure S8C). Downregulated DEGs were uniquely enriched with the cell signaling part of the biological pathway (Figure S8D). Additionally, DEGs were enriched with the cell cycle (Figure S9A), catabolic process (Figure S9FB), plasma membrane component (Figure S9C), mitochondria metabolic pathway (Figure S9D), apoptosis (Figure S9E), and p53 pathway (Figure S9F).

#### 3.3.2 Aberrant Expression of Genes Encoding Transcription Factors, Collagen Synthesis, Cytokine, Kinase, Insulin Signaling Pathway, and Oncogenic Genes in the Ovary

**Figure 3:**
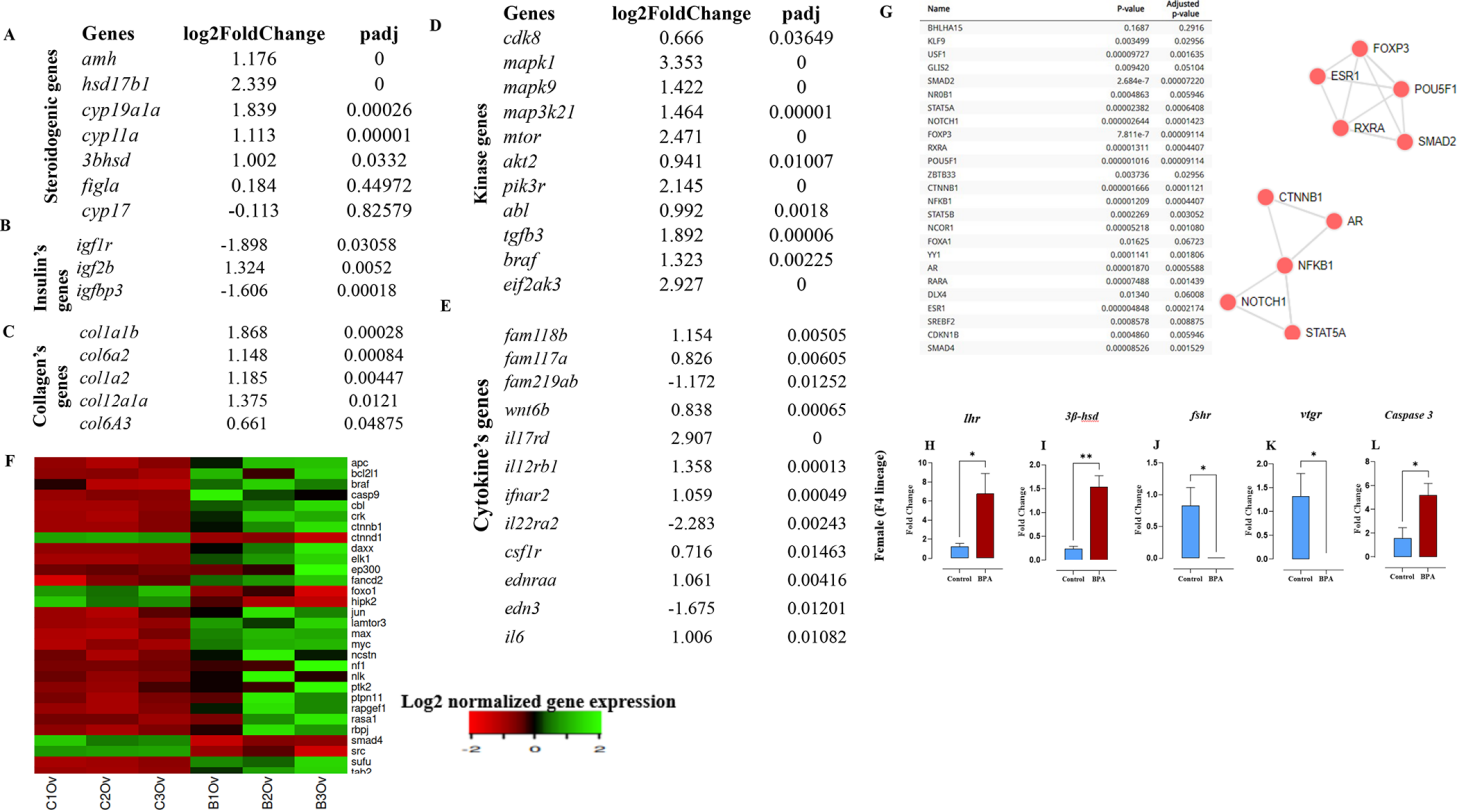
Abnormality in the expression of genes encoding (A) steroidogenic metabolism, (B) insulin signaling, (C) collagen synthesis, (D) kinase, (E) cytokine, and (F) cancerous genes. (G) List of significant transcription factors and their network in the ovary of the BPA lineage fish. Transcriptional alterations in gonadotrophin receptor genes in the ovaries of the BPA lineage fish. A significant increase in (H) *lhr* and (I) *3*β*-hsd* transcripts was observed in the BPA lineage ovaries. A significant decrease in (J) *fshr* and (K) *vtgr* transcript levels was found in the BPA lineage. A significantly abundant transcript of (L) *caspase 3* was measured in the BPA lineage ovaries. Asterisk indicates statistical significance at the level of *P < 0.05, *t* test compared against control lineage.

Expression patterns of the genes that are associated with ovarian steroidogenic functions, including *amh*, *hsd17b1*, *cyp19a1*, *cyp11a1*, *3*β*-HSD*, and *cyp17*, were examined. RNA-seq results showed a significant upregulation of *Cyp11a1*, *amh*, *hsd17b1*, *3*β*-HSD*, *Cyp19a1* and a significant downregulation of *cyp17* in the BPA lineage group (Figure 3A). The expression of *igf1r* and *igfbp3* was downregulated, whereas *igf2b* expression was significantly upregulated, suggesting abnormal insulin signaling in the ovary (Figure 3B). The expression of genes encoding collagen proteins, mainly *col1a1b*, *col1a2*, and *col12a1*, was significantly upregulated (Figure 3C). This suggests abnormal tissue deposition in the ovary of the BPA linage. In the BPA lineage, specific genes encoding kinases, including *mapk1*, *mapk9*, *mtor*, *pik3r*, and *eif2ak3*, and cytokines, including *il17rd*, *il12rb1*, and *fam118b*, were significantly upregulated. However, the expressions of *il22ra2*, *fam219ab*, and *edn3* were significantly downregulated (Figure 3D,E). Abnormal expression of gene-encoding kinase and cytokine indicates molecular pathogenicity. The expressions of genes involved in malignancy such as *myc*, *akt1*, *apc*, *mtor*, *bcl2l1*, *daxx*, and *jun* were significantly upregulated, and only *smad4* and *src* genes were downregulated in the ovary of the BPA lineage group (Figure 3F). Additionally, essential transcription factors, such as *esr1*, *foxp3*, *smad 2*, *ar*, nfkb1, nothch1, klf9, and stat5 involved in the disease pathway were also identified (Figure 3G).

#### 3.3.3 Alterations in the Expression of Reproductive Genes, Apoptosis Genes, and Genes Encoding Vitellogenin Receptors in the Ovary of the BPA Lineage

The mRNA levels of *lhr* (*P* < 0.05, Figure 3H) and *3*β*-hsd* (*P* < 0.01, Figure 3I) were significantly increased in the ovary of the BPA lineage. However, mRNAs of *fshr* (*P* < 0.05, Figure 3J) and *vtgr* (*P* < 0.05, Figure 3K) were significantly decreased in the ovary of the BPA lineage group. This indicated transgenerational dysregulation of steroidogenesis and reproduction-related pathways in the BPA lineage fish. Additionally, the levels of *caspase3* mRNAs were significantly increased (*P* < 0.05, Figure 3L) in the ovary of the BPA lineage, suggesting increased apoptosis-mediated cell death in the ovary of the BPA lineage fish.

#### 3.3.4 DEGs of the F4 BPA Lineage PCOS Fish Overlapped with Those of the Human PCOS Patients

**Figure 4:**
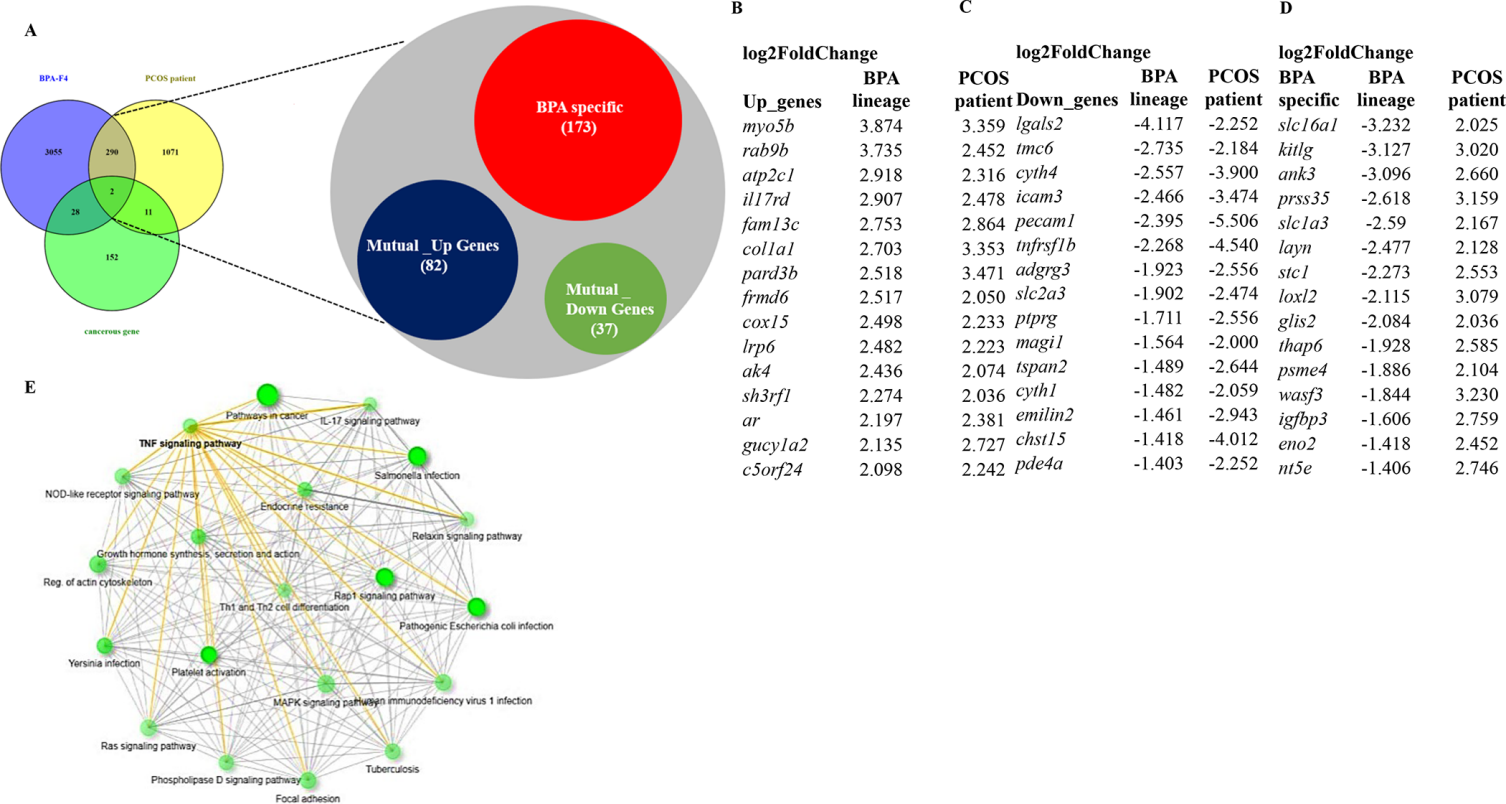
Comparison of DEGs found in the PCOS patient data set with those in the ovaries of BPA lineage fish. (A) An intersection of the Venn diagram showing common DEGs. (B) Top 15 mutually upregulated DEGs. (C) Top 15 mutually downregulated DEGs. (D) Top 15 mutual BPA-specific DEGs and (E) KEGG pathway enrichment on common DEGs.

DEGs of BPA lineage PCOS fish were compared with publicly available human PCOS patient DEGs to identify end-point specific (PCOS) DEGs associated with ancestrally induced PCOS (end point) in the F4 generation.^66^ In total, 292 common DEGs were found from the total of 1374 DEGs found in the human PCOS patient data set (Figure 4A). Additionally, 82 DEGs were mutually upregulated in both BPA lineage and PCOS patients. Among the total shared DEGs in BPA and PCOS patient groups, 37 DEGs were mutually downregulated. However, 173 shared DEGs showed exclusive expression patterns in the BPA lineage (Figure 4A). From the total shared DEGs, the top 15 mutually upregulated (Log2FC > 2), downregulated (Log2FC < −1), and exclusive BPA-specific DEGs are illustrated (Figure 4B–D). Heatmaps show shared common total DEGs between BPA lineage and PCOS patients (Figure S10A–D). The comparative analysis revealed that the BPA lineage ovaries have similar gene expression patterns to human PCOS patients. KEGG pathway analysis on 292 DEGs showed significant enrichment in TNF signaling, IL signaling, growth hormone synthesis, and regulation of the actin cytoskeleton (Figure 4E). The transcriptome database has been submitted to NCBI as GSE226322 (token # qtqrqoycjtqfxqb).

#### 3.3.5 IPA and Upstream Regulator Analysis

**Figure 5:**
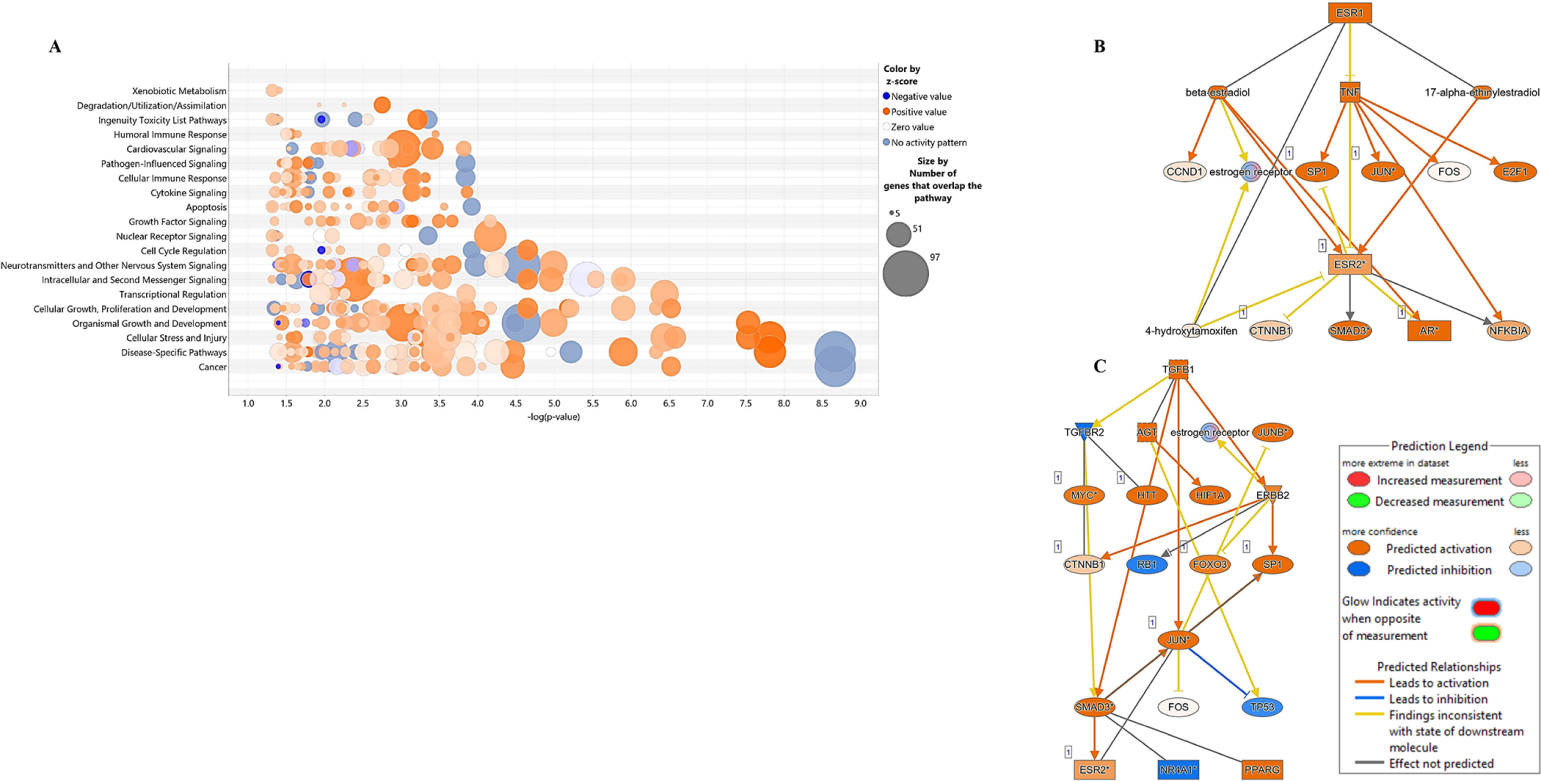
IPA analysis on the DEGs in the BPA lineage ovaries. (A) Canonical pathway. Upstream regulator gene networks within the DEGs determined by IPA. (B) ESR1 and (C) TGFB1 with interactive networks with downstream genes in the development of ovary pathogenesis.

To further understand the biological pathway involved in diseased phenotype induced by ancestral BPA exposure, the RNA-seq data were annotated into human IDs for core functional analysis using IPA. Canonical pathways enriched in the molecular mechanism of cancer (*P* value 2.15E-09), autophagy (*P* value 2.95E-08), HOTAIR regulatory pathway (*P* value 3.00E-07), cellular stress and injury, cell cycle and transcriptional regulation, apoptosis, cytokine signaling, and immune response were determined (Figure 5A). DEGs were involved in the molecular mechanism of cancer (Figure S11), autophagy (Figure S12), and HOTAIR (Figure S13). Additionally, the gene-disease network analysis showed that *akt1* and *tnf* are master regulators of various disease pathways, such as HOTAIR regulatory pathways, AMPK signaling, growth failure, and autophagy, which are all indicative of severe ovarian disease due to ancestral BPA exposure (Figure S14). Using the upstream regulator analysis (URA) tool, IPA can identify potential upstream regulators by analyzing linkage to DEGs via coordinated expression.^83^ A total of 94 transcription regulators were identified, and the top 15 transcription regulators were determined (Figure S15). e*sr1* (estrogen receptor1) and *tgfb1* (transforming growth factor beta 1) were the most significant upstream regulators interacting with several genes such as *jun*, *myc*, *foxo3*, *smad3*, and *e2f1* (Figure 5B,C).

### A Shift in Global Metabolites and Pathway Enrichment in the Ovary

A

**Figure 6:**
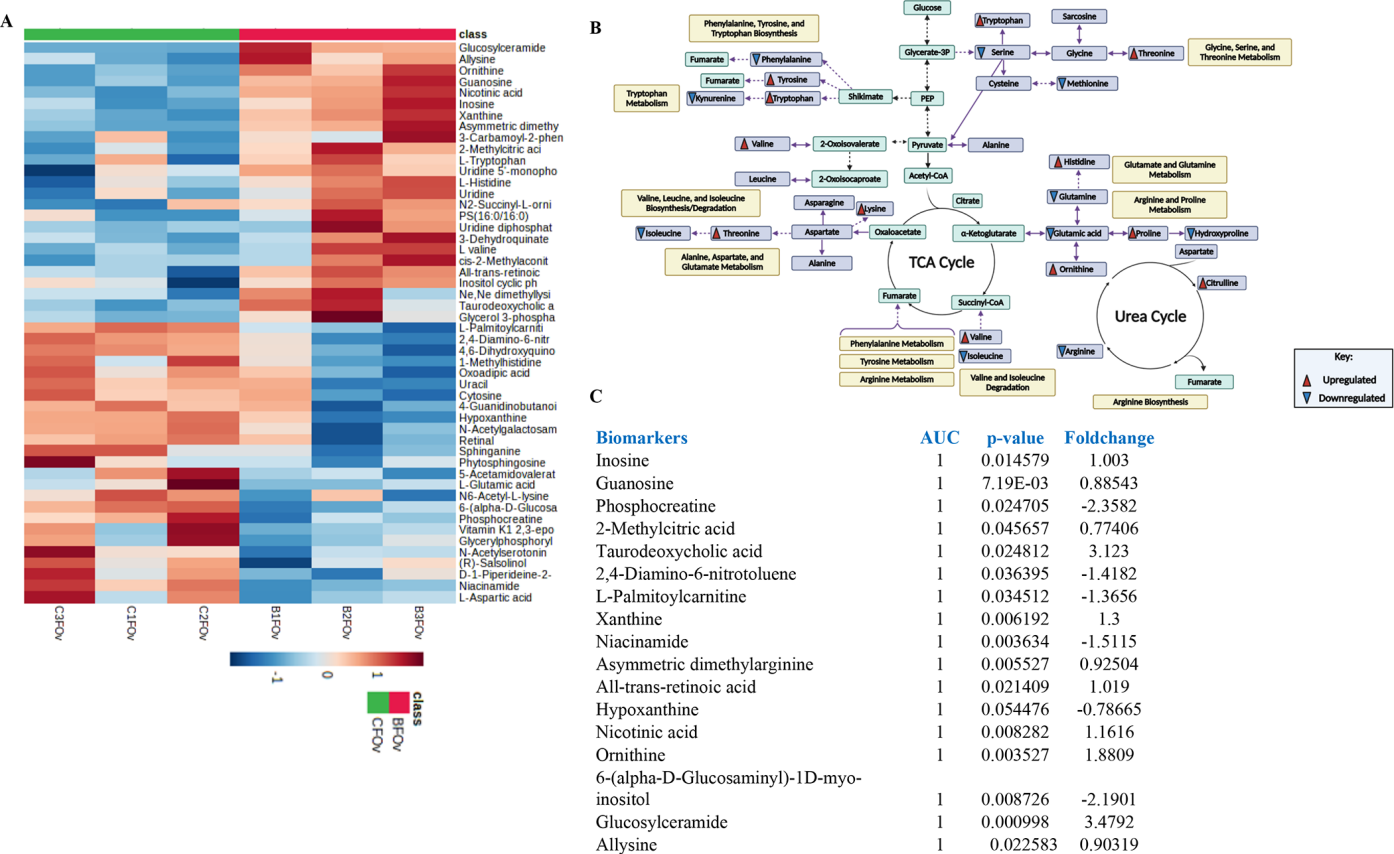
Global metabolic alterations in the ovary of BPA lineage fish. (A) Heatmap showing a differential abundance of the top 50 significant metabolites. (B) The abundance profile of amino acids in the PCOS metabolic pathway. (C) Table of biomarkers and the list of ovarian metabolites.

Metabolomic analysis was conducted to address the alterations in ovarian metabolic profiles due to ancestral BPA exposure effect in the BPA-exposed control lineage group. An orthogonal partial least square discriminant analysis (OPLS-DA) was performed to elucidate the differences between the BPA and the control lineage group. According to the score plot, there was a difference among the groups (Figure S16). In this OPLS-DA model, R2X = 0.29, R2Y = 0.91, Q2 = 0.5. Metabolites with VIP > 1 are shown in Figure S17 with *m*/*z*, retention time, and HMDB class and IDs. The clustered heatmap showed the top 50 significant metabolites and their relative abundance (Figure 6A), providing useful insights into the relationship between unique metabolites of the BPA lineage and control lineage. The metabolites belonged to amino acids, purine and pyrimidine derivatives, organic acids, lipid molecules, sugars, and some other class of molecules. Lipid molecules such as glucosylceramide; PS (16:0/16:0); amino acids mainly l-histidine, allysine, l-tryptophan, and l-valine; and purine derivatives mainly guanosine, xanthine, hypoxanthine, and guanine were found in higher abundance in BPA lineage group. 4,6-Dihydroxyquinoline, vitamin K1 2,3-epoxide, uracil, phosphocreatine, cytosine, and l-aspartic acid were detected but in low abundance. Differential abundance of amino acids from the ovary of the BPA lineage fish was mapped in metabolic pathways associated with PCOS (Figure 6B). Biomarkers of PCOS were found in the ovary metabolite data. With *P* < 0.05 and AUC = 1, a total of 17 biomarkers were found in the ovary of the BPA lineage (Figure 6C). Those were inosine, guanosine, phosphocreatine, 2-methylcitric acid, taurodeoxycholic acid, 2,4-diamino-6-nitrotoluene, l-palmitoylcarnitine, xanthine, niacinamide, asymmetric dimethylarginine, all-*trans*-retinoic acid, hypoxanthine, nicotinic acid, ornithine, 6-(alpha-D-glucosaminyl)-1D-myo-inositol, glucosylceramide, and allysine (Figure S17). The top 25 highly enriched metabolic pathways were identified. Metabolically important pathways such as arginine biosynthesis, aminoacyl-tRNA biosynthesis, histidine metabolism, proline metabolism, and glutathione metabolism were highly enriched in the BPA lineage ovary (Figure S18).

#### 3.4.1 Comparative Analysis of the Metabolites of BPA Lineage Fish Ovary with PCOS Patient Metabolite Data Set

**Figure 7:**
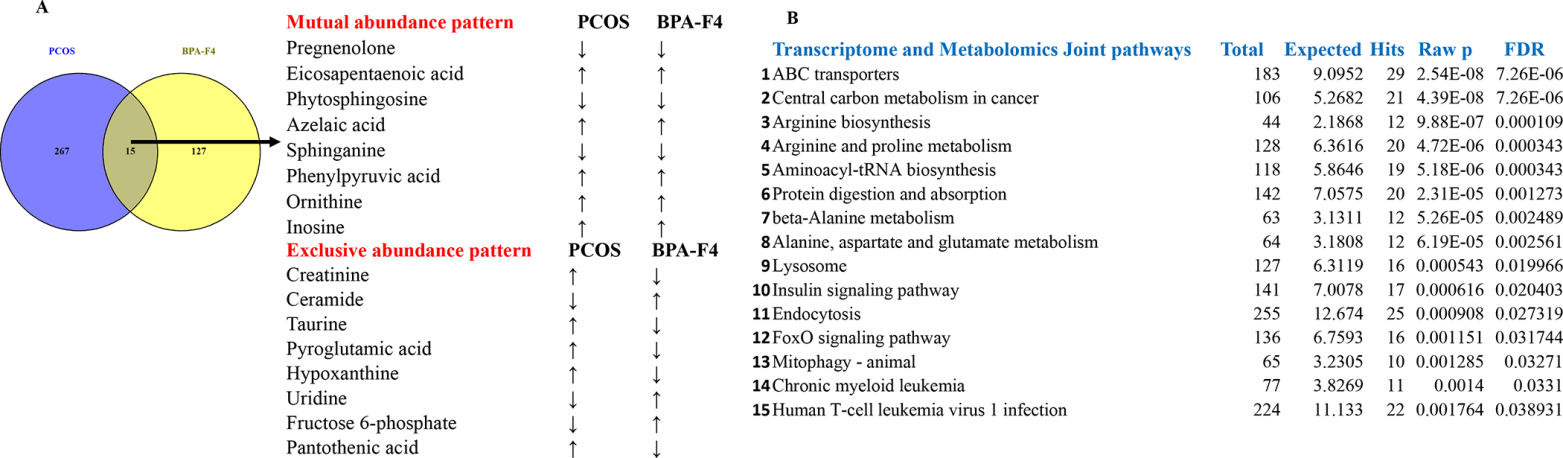
Comparison of metabolites between the PCOS patient data set and the ovary of BPA lineage fish. (A) A Venn diagram showing overlapped metabolites in both the PCOS patient data set and the ovary of BPA lineage fish. (B) Joint pathway analysis of transcriptome and metabolomes with list of the pathways with total hits, *P* value, and FDR.

To investigate potential metabolites linked to transgenerational PCOS, the metabolites of F4 ovary from BPA lineage were compared with the PCOS patient metabolite data.^81^ A total of 16 metabolites were found to be common between BPA lineage and PCOS patients (Figure 7A). Among the 16 metabolites, 8 common metabolites showed a mutual pattern of abundance, and 7 of them showed an exclusive abundance pattern in the ovary of BPA lineage. Pregnenolone, eicosapentaenoic acid, phytosphingosine, azelaic acid, sphinganine, phenylpyruvic acid, ornithine, and inosine showed similar abundance patterns in the ovary of BPA lineage and PCOS patient data set (Figure 6A). However, creatinine, taurine, uridine, and taurine exhibited an exclusive pattern of abundance under common metabolites (Figure 6A). KEGG pathway analysis was conducted on common metabolites found in the ovary of BPA lineage and human patients. These pathways included sphingolipid metabolism, glutathione metabolism, phenylalanine, tyrosine and tryptophan metabolism, taurine metabolism, and hypotaurine metabolism (Figure S18).

#### 3.4.2 Integrated Metabolomic and Transcriptomic Pathway Analysis Revealed Deleterious Pathways in the Ovary of BPA Lineage Fish

Significantly altered metabolites and genes were submitted to joint pathway analysis in Metaboanalyst.^84^ Out of the 324 biological processes identified, 74 pathways had *P* value < 0.05 and FDR < 25%. The top three pathways based on the calculation of *P* value were the ABC transporter, carbon metabolism in cancer, and arginine biosynthesis (Figure 7B). The gene–metabolite interaction network and metabolite–metabolite interaction network are shown in Figure S19. Metabolites found in carbon metabolism for triggering cancer were mapped in Figure S20. The insulin signaling, endocytosis, and cancer-related pathways that were found prominent in both omics were also significantly enriched in the integrative analysis.

### 3.5 Abnormal Fat Accumulation and Hepatosteatosis in the Liver of the F4 Females

**Figure 8:**
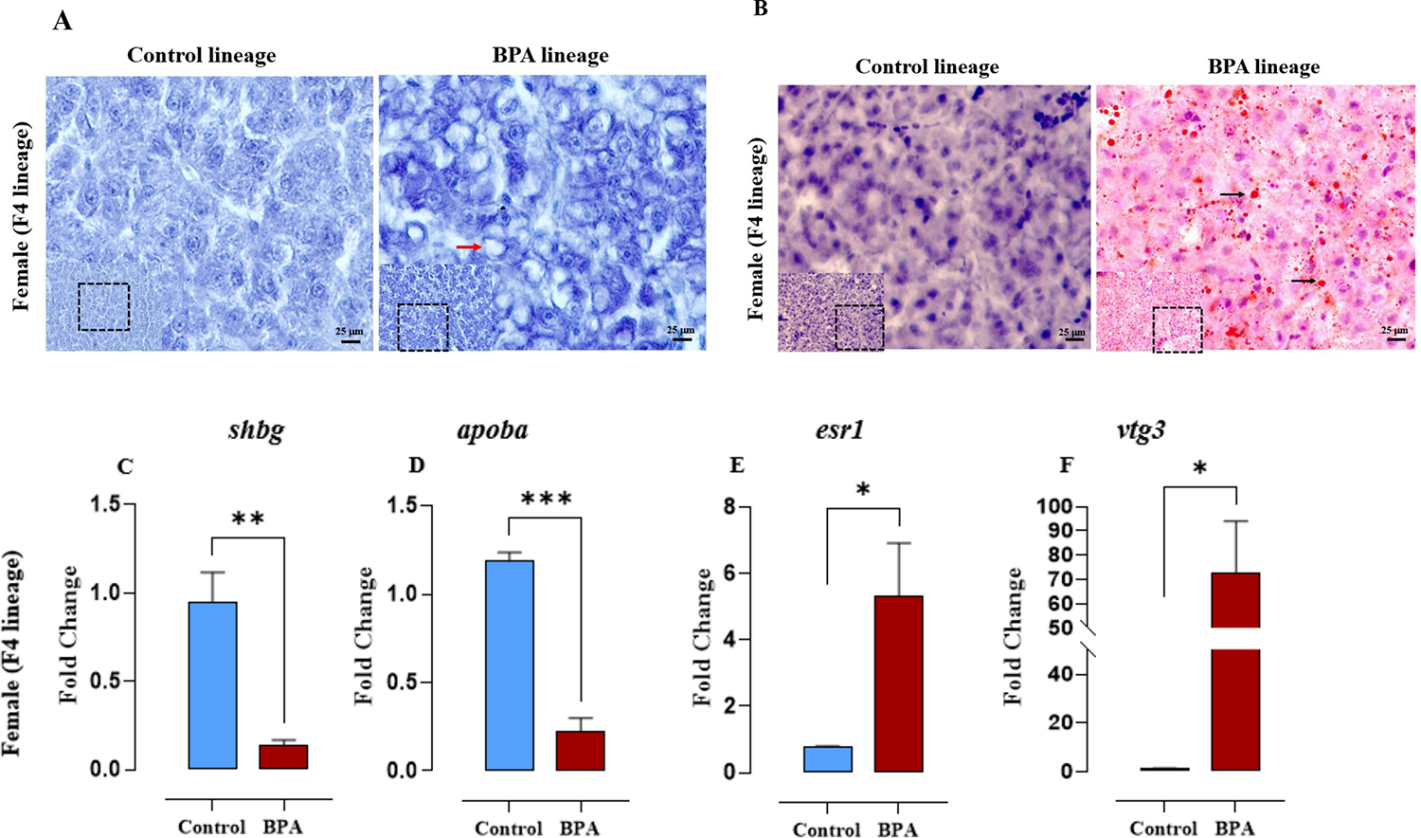
Ancestral BPA exposure resulted in liver steatosis in females of the F4 generation. (A) Liver histology micrograph. (B) Fat accumulation in the liver of the F4 generation females (magnification ×40). Transgenerational alterations in the expression of genes in BPA lineage livers. Significant decrease in (C) *shbg* and (D) *apoba* transcript in BPA lineage fish. Significant decrease in the levels of (E) *esr1* and (F) *vtg3* transcripts in BPA lineage fish. Asterisks indicate statistical significances at the level of *P < 0.05 and ***P < 0.001 compared to control lineage by *t* test.

The liver in the BPA lineage females had more vacuolated hepatocytes than in the control lineage fish (Figure 8A). The hepatic cells were arranged in sheets separated by sinusoidal meshes. To determine if ancestral BPA exposure caused lipid accumulation in the liver, which is also a symptom of NAFLD, Oil Red O was used to stain hepatic neutral lipids. Consistent with their higher hepatosomatic index (HSI), cryosections of the livers of the BPA lineage females showed a proportional accumulation of neutral lipids (Figure 8B), indicating the liver of BPA lineage developing NAFLD.

### 3.6 Expression of Vitellogenic and Sex Hormone Binding Globulin Genes in the Liver

To examine whether ancestral BPA exposure altered the expression of vitellogenic genes in the F4 generation liver, expression profiles of the vitellogenic genes were determined by qPCR. The mRNA levels of the sex hormone binding globulin (*shbg*) gene, a PCOS marker,^85^ were significantly decreased in the BPA lineage livers (Figure 8C). Furthermore, the expression of genes involved in vitellogenin synthesis pathway in the liver was significantly altered. Mainly, the expression of apolipoprotein ba (*apoba*) was significantly downregulated (Figure 8D), and both estrogen receptor 1 (*esr1*) and vitellogenin 3 (*vtg3*) were significantly upregulated (Figure 8E,F) in the BPA lineage.

## 4 Discussion

According to familial clustering and twin studies, PCOS is an inherited disease.^86,87^ Given that approximately 10% of its human PCOS loci are genetic and inherited,^88^ the overall pathogenesis of PCOS suggests its onset and regulation by environmental and nongenetic mechanisms. Environmental chemical exposures during germ cell reprogramming can leave chemical-specific epigenetic marks on germ cells, which result in phenotypic abnormalities that persist through several generations.^89−91^ When directly exposed to BPA, females develop metabolic disorders or PCOS in the immediate generation (F0).^92−95^ However, it is not clearly understood whether future-generation females can still suffer metabolic and reproductive diseases even after the withdrawal of BPA from consumer products and the environment. This study demonstrates for the first time using medaka fish as an animal model that embryonic exposure to BPA during germ cell reprogramming in a grandparental generation leads to multidisease transgenerational phenotypes, PCOS, and NAFLD in the fifth generation who did not experience BPA exposure. The hypothalamus–pituitary–gonad (HPG) axis in fish resembles that in mammals, suggesting a great potential to study human PCOS as an alternative nonmammalian model.^36,37,40^ Medaka and zebrafish utilize similar pathways of sex hormonal regulation of steroidogenesis but process epigenome in their embryo and germline cells differently.^39^ Unlike other fish species, medaka processes epigenetic information in postfertilization embryos and primordial germ cells similar to humans and mice, making them excellent transgenerational models.^38,39^ This suggests that the mechanistic molecular information obtained from medaka could be valuable for understanding ancestral epigenetic effects of BPA in the development of PCOS in the subsequent generation of other higher vertebrates, including humans.

In the present study, F4 females of the BPA lineage group displayed abnormal metabolic traits, such as increased BMI and visceral fat accumulation. The ability of BPA to induce fat accumulation has been demonstrated in a direct exposure model. BPA caused insulin resistance and disrupted lipid homeostasis in primary human preadipocytes, resulting in abnormal fat accumulation *in vitro*,^96−100^ suggesting BPA’s ability to modulate fat deposition due to chronic exposure. However, how visceral fat deposition occurs in unexposed generations due to ancestral exposure is unclear. In addition to transgenerational fat deposition in the BPA lineage group, significant upregulation of the expression of *il6* and genes encoding several cytokines and downregulation of *igf1* were found in the ovary of the BPA lineage, indicating a potential state of insulin resistance mediated by visceral fat accumulation. Congruent with a previous study, an altered expression pattern, particularly the upregulation of *il6* and activation of *tnf-*α, a master regulator, was found in the ovary of BPA lineage, indicating visceral fat mediation due to the activation of proinflammatory cytokines (*TNF-*α, *IL-6*) and tissue macrophages, critical drivers of PCOS-obesity multifactorial disease.^101,102^ The downregulation of *igf1* in the ovary of the BPA lineage could also have contributed to impaired follicular growth, as reflected histologically. The literature shows decreased levels of IGF-I and IGF-II proteins in the follicular fluid of PCOS women as well as reduced expression of IGF-1 receptor in human granulosa cells,^103,104^ suggesting the role of IGF protein in PCOS development. The present observations provide an additional line of information that environmentally induced transgenerational fat deposition could have activated cytokines and decreased follicular maturation in females of the BPA lineage, leading to PCOS phenotype.

The gonadotropins play a vital role in PCOS pathogenesis, and in the teleost, they regulate the vitellogenesis and final oocyte maturation/ovulation.^105,106^ In the present study, lower levels of *fshr* mRNA and higher levels of *lhr* mRNA were detected in the ovary of BPA lineage fish than in the control. In teleost, gonadotropin receptor expression positively correlates with plasma hormone levels.^107^ The present results indicated lower levels of mRNAs for FSH and higher levels of LH in the ovary of BPA lineage fish, suggesting transgenerational hormonal imbalance. A high LH/FSH ratio is a characteristic of PCOS patients. Similar patterns of gonadotropin receptor gene expression suggest a similar trend.^108^ Increased levels of plasma FSH and ovarian follicle-stimulating hormone receptor (*FSHr*) mRNA levels are required for the transition of oocytes from the previtellogenic stage (stage II) to vitellogenic stages (stage III) during ovarian maturation.^106,109,110^ In the present study, abnormal follicular development in stage II (previtellogenic oocyte) was found, along with the downregulation of *fshr* in the ovary of BPA lineage. Ovarian follicles are arrested at the primary growth-previtellogenic transition in zebrafish mutant for *fshr.*(111) The restricted maturation of follicles observed in the ovary of BPA lineage medaka that leads to anovulation could be due to deficiency of *fshr*, which is a primary phenotype for PCOS.^112^ Consistent with our present histopathological findings in medaka, similar histological phenotypes were found in the rat model of PCOS, suggesting that the BPA lineage group had developed PCOS phenotype in the ovary.^113^ Direct exposure to BPA has been linked to follicular arrest and atresia leading to anovulation,^114−116^ suggesting that transgenerational PCOS could be driven by mechanisms similar to those reported from direct exposure studies. The critical yet undiscovered information is delineating heritable molecular determinants of PCOS and developing strategies to block pathways to disease, as environmental BPA exposure (past and present) might already have left such molecular memories in the germline of the existing population.

The development of PCOS is intrinsically linked to steroidogenic dysregulation. The upregulation of androgenic genes such as *3*β*-hsd*, *hsd17b1*, and *cyp11a* in the ovary of BPA lineage medaka could have potentially enhanced testosterone biosynthesis in ovarian theca cells. This can be validated by a human study in which a positive association of increased free testosterone (hyperandrogenemia) was found with increased visceral fat deposits in women with PCOS.^117^ This clearly suggests that transgenerational deposition of visceral fat could be due to the biosynthesis of testosterone in BPA lineage females. In contrast, the expression of C*yp19a1a*, the gene encoding estrogen-synthesizing enzyme aromatase, was increased in the ovary of the BPA lineage females. The fold change of androgenic genes was more significant than that of estrogen-synthesizing genes, indicating an increase in the hypothetical ratio of androgen/estrogen, a characteristic of PCOS.^118^ An *in vitro* study found that hyperactivation of *lhr* increases adenylate cyclase, which triggers the synthesis of *3*β*-hsd.*^119,120^ Increased levels of LH boost the synthesis of androgen from theca cells of the ovary leading to ovarian dysfunction.^121,122^ Congruent with our observation, the upregulation of *lhr* expression could have induced the upregulation of *3*β*-hsd*, enhancing androgen synthesis in the ovary. As a result, dysregulation of the steroidogenic pathway could have contributed to potential reproductive impairment related to the PCOS phenotype in the F4 BPA lineage fish.

PCOS-specific DEGs activated in the ovary of BPA lineage fish were compared with the PCOS patient transcriptome data set. In total, 292 common significant DEGs were determined, potentially contributing to pathogenesis, including genes associated with activating the TNF signaling pathway, endocrine resistance, and cancer pathway. Consistent with GSEA analysis, IPA revealed canonical pathways such as autophagic mechanism, cell cycle regulation, cellular stress and injury, and cancer pathway with the activation of AKT1 and TNF. Interestingly, genes associated with activating the cancerous pathway, i.e., *myc*, *akt1*, *apc*, and *mtor*, were upregulated in the ovary of the BPA lineage group. Alternatively, the ovary of the BPA lineage group showed decreased expression of *foxo1* and tumor suppressor gene (*smad 4*) genes. Upregulation of c-MYC, enhanced AKT activity, activated protein C (APC), and reduced FOXO3a activity were observed in epithelial ovarian carcinoma.^123−126^ In line with a previous study conducted by Bornstein *et al.* showing that a loss in SMAD4 increases genomic instability associated with the TGF-β signaling in ovarian cancer, increased expression of *tgf-*β in the ovary of BPA lineage was detected, suggesting an advanced stage of ovarian disease.^127^ Among the top 10 upregulated genes, three genes, namely, *selenop*, *RNA-nf1*, and *olfml3*, were associated with sex-hormone modulation^128^ and found in PCOS follicular fluid,^129^ controlling cell cycle progression associated with ovarian teratoma.^130^ In the top 10 downregulated genes, *fabp6* and *arl6* (ADP ribosylation factor like GTPase6) were involved in ovarian disease^131^ and modulating membrane trafficking and cytoskeletal function.^132^ As transcriptional dysregulation and diseases are influenced by chromatin organization,^133^ we were interested in deciphering epigenetic gene expression controlling the transcriptional output of the cell. Among the 91 DEGs found to be involved in chromatin organization, *kdm2b*, *ehmt1*, *hdac4*, *kmt2d*, *jmjd6*, *ctcf*, *setd5*, *suz12*, *btaf1*, and *bahd1* were all upregulated, whereas *dnmt3ba* and *dnmt3b* were downregulated, indicating transgenerational alteration of epigenetic genes associated with PCOS phenotype in the ovary of BPA lineage. Previous studies showed that upregulation of KDM2B and HDAC and dysregulation of EHMT1/2 are linked to DNA damage and dysregulation of the cell cycle, and overexpression of *HDAC* is linked to ovarian cancer.^134,135^ Together with the published literature, differential expression of PCOS-specific genes and epigenetic genes linked to histone modifications and DNA methylation indicates a role of epigenetic genes associated with PCOS phenotype in the ovary of BPA lineage fish.

In the ovary of the BPA lineage fish, several transforming growth factors (TGFs) were differentially expressed, indicating activation of several downstream genes such as fibrotic genes such as *col1a1b*, *cola2*, *col2a1a*, *jun*, *foxo3*, *sp1*, and *olfml3* and extracellular matrix protein genes. In addition, the expressions of *hspa5* and *eif2ak3* (unfolded protein response (UPR) genes) were upregulated in the BPA lineage ovary compared to control, indicating that ER stress response could have potentially activated the TGF β pathway.^136^ The ovary of the BPA lineage fish showed significant upregulation of several transforming growth factors, including upstream regulators *tgfb1* and *tgfb3*, indicating the activation of several downstream fibrotic genes such as *col1a1b*, *cola2*, *col2a1a*, *jun*, *foxo3*, and *sp1*. The ovary of the BPA lineage fish also showed extracellular tissue deposition in accordance with the activation of fibrotic genes and extracellular matrix protein gene *olfml3*. Expression of genes related to ER stress, UPR, and TGF-1 was upregulated in granulosa cells of the ovary and was linked to tissue fibrosis.^137−140^ Extracellular matrix accumulation in the form of collagen deposition in the ovarian capsule of the BPA lineage is linked to PCOS pathogenesis.^141^ Using GSEA analysis, 94 DEGS were identified as associated with autophagy, including upregulated *atg3*, *becn2*, and *casp3* and downregulated foxo1 genes, suggesting aberrant autophagic mechanism activated in the ovary of the BPA lineage. The literature suggests that ATG3 and Foxo1 levels are downregulated in PCOS patients and that decreased levels of autophagy markers (ATG7 and BECN1) directly inhibit oocyte maturation.^142,143^ Moreover, an elevated *caspase3* expression in the ovary, which is a biomarker for PCOS, can trigger aberrant apoptosis resulting in the development of cysts resulting from degenerated follicles found in the ovary of the BPA lineage.^144^ Direct exposure to BPA has been found to activate proinflammatory cytokines IL6 and TNFα that are involved in fibrosis.^145^ However, in transgenerational PCOS ovaries, activation of the TGF signaling pathway and ER stress response was synergistically involved in fibrosis induced by ancestral BPA exposure.

Compared with the previous metabolomic studies on follicular fluid from PCOS patients, the changes in several metabolites in the ovary of BPA lineage were consistent with PCOS patients.^81^ Mutual metabolites were significantly enriched in sphingolipid metabolism, glutathione metabolism, taurine, hypotaurine metabolism, and phenylalanine metabolism. An abnormal expression of sphingolipids such as lysophosphatidylcholines and phosphatidylethanolamines was found to be associated with PCOS.^82^ Physiological deviations in amino acid concentrations may lead to pathogenic conditions, such as oxidative stress and ovarian disease, as well as metabolic disturbances, such as type 2 diabetes, obesity, and insulin resistance.^146−148^ A high abundance of valine, histidine, tryptophan, and creatinine was found in the BPA lineage group. In agreement with our finding, an analysis performed by Zhao *et al.* found that the levels of alanine, valine, serine, threonine, ornithine, phenylalanine, tyrosine, and tryptophan are generally increased and the levels of glycine and proline are reduced in plasma samples of PCOS patients.^82^ In the present study, PCOS-specific pathways, including arginine biosynthesis,^149,150^ aminoacyl-tRNA biosynthesis,^151,152^ beta-alanine metabolism,^150^ proline and arginine metabolism,^153,154^ taurine metabolism,^155^ and phenylalanine metabolism,^150^ were activated in the ovary of the BPA lineage group, suggesting that metabolic alterations were promoted by ancestral BPA exposure effect.

The present results from a human–fish comparative metabolomics–transcriptome analysis show distinctly altered ABC transporter, central carbon metabolism in cancer, aminoacyl-tRNA biosynthesis, protein digestion, and absorption pathways, indicating that BPA has a potential role in the development of PCOS in fish and humans. Changes in the ATP binding cassette transporter 1 (ABC1) gene, which encodes the protein regulating entry and exit from the cell membrane, may contribute to dyslipidemia in patients with PCOS.^156,157^ In addition, the top five significantly enriched KEGG pathways for PCOS vs premature ovarian follicle (POF) group metabolites included protein digestion and absorption pathways, ABC transporter-dependent pathways, central carbon metabolism pathways in cancer, aminoacyl-tRNA biosynthesis pathway, and prostate cancer pathways found in human studies.^158^ Additionally, arginine proline metabolism, insulin signaling pathway, and lysosomal pathways were found to be involved in ancestral BPA exposure-induced PCOS. A KEGG pathway analysis indicated that insulin signaling, MAPK signaling, AMPK signaling pathway, and autophagic pathway were enriched by total upregulated genes, whereas Rap1, cAMP, and AGE-RAGE signaling pathways were enriched by total downregulated genes, indicating PCOS-specific pathways in the ovary of the BPA lineage. Activation of the Rap1 signaling pathway is found in cancer, and AGE-RAGE signaling is involved in the pathogenesis of diabetes.^159,160^ Several studies have shown that PCOS is associated with impaired insulin signaling, aberrant MAPK signaling, and abnormal AMPK signaling in ovarian cumulus and granulosa cells.^66,161,162^ The present results, together with the published literature on PCOS development and progression, show that ancestral BPA exposure seems to have activated deleterious signaling pathways in the ovary of the BPA lineage leading to advanced-stage ovarian disease. The PCOS phenotype was observed in females that developed severe NALFD.

The presence of low *apoB* transcript in the liver of BPA lineage females was accompanied by significant fat accumulation, suggesting that a lack of fat molecules released by the liver may have contributed to abnormal fat accumulation, as indicated by the oil and red stain in the liver. Downregulation of *apoB* isoforms may result in low levels of TGs and cholesterol in the circulation as well as an extensive accumulation of lipid droplets in hepatocytes, indicating the development of NAFLD phenotype.^163^ In BPA lineage females, the levels of *shbg* mRNAs were significantly decreased in the liver, suggesting another possible link between transgenerational PCOS and NAFLD. The PCOS biomarker SHBG protein is a circulating homodimeric glycoprotein synthesized by hepatocytes with a stronger affinity for testosterone than estrogen.^164^ The expression of *shbg* was decreased in the liver of the BPA lineage females. Such a decrease in expression has been found to positively correlate with the bioavailability of free testosterone,^165^ hyperandrogenism,^166^ fatty liver disease, hyperinsulinemia, and PCOS. These observations reflect a cross talk between the hepato-ovarian axis in BPA lineage fish. Additionally, the F4 generation of the BPA lineage group showed highly upregulated *esr1* and *vtg3* transcripts in the liver, indicating estrogen-mediated induction of vitellogenin synthesis that enhances fat body synthesis in the liver.^167^ However, the uptake of vitellogenin via the vitellogenin receptor in the ovary is highly important for the maturation of oocytes in teleost to maintain a steady state of follicular balance.^168^ In the BPA lineage fish, vitellogenin receptor (*vtgr*) expression was significantly reduced, suggesting a perturbed vitellogenin uptake that could have prevented the transition from previtellogenic to vitellogenic oocytes and promoted the immature follicular state related to the PCOS phenotype in the BPA lineage fish.

The present results demonstrate a multidisease phenotype of NAFLD–PCOS in females four generations after direct embryonic BPA exposure at F0 generation. At this time of embryonic development, the liver and gonadal germline cells are differentiating from their progenitor cells. Results suggest that BPA exposure not only causes PCOS in an immediate generation but also can cause multidisease phenotype in offspring after several generations, via germline transmission. Integration of both transcriptomic and metabolomic results revealed the presence of affected metabolic routes in the ovary of the BPA lineage, suggesting a potential mechanism underlying transgenerational reproductive impairment. Together with the transgenerational inheritance concept of the published literature, the present study suggests that ancestral BPA exposure-induced molecular memories, not yet discovered, may be promoting these multidisease phenotypes. Environmentally established molecular memories are transferred from the germline to the somatic cells (e.g., liver and ovary).^7^ To demonstrate causative relationships between germline epigenome and observed transgenerational phenotypic traits in the ovary and liver, it is imperative to screen the ancestral germline epimutations induced by BPA and correlate them with epigenomes of liver and ovary of subsequent generations and their manipulation by CRISPR-mediated epigenome editing to understand their role in the development of multidisease phenotypes.

## Supporting Information Available

A global alteration in gene expression in the ovary of the BPA lineage exhibited by heatmap (Figure S1); Gene Ontology analysis in the ovary of BPA lineage (Figure S2); DEGs enriched in the insulin signaling pathway in the ovary of BPA lineage (Figure S3); DEGs found in the MAPK signaling pathway in the ovary of BPA lineage (Figure S4); DEGs enriched in the AMPK signaling pathway in the ovary of BPA lineage (Figure S5); DEGs found in the Rap1 signaling pathway in the ovary of BPA lineage (Figure S6); DEGs found in the cAMP signaling pathway in the ovary of BPA lineage (Figure S7); standard GSEA analysis and heatmap of enriched DEGs associated with pathogenesis (Figures S8 and S9); the PCOS-specific mutual DEGs in the ovary of the BPA lineage fish and PCOS patient data set (Figure S10); molecular mechanism of the cancer triggering pathway in the ovary of the BPA lineage fish (Figure S11); molecular mechanism of the autophagy triggering pathway in the ovary of the BPA lineage fish (Figure S12); molecular mechanism of the HOTAIR mechanism in the ovary of the BPA lineage fish (Figure S13); gene disease network via Ingenuity Pathway Analysis (IPA) showing activation of *akt1*, *tnf*, and *ifng* associated with activation of disease specific pathways (Figure S14); predicted top 15 upstream regulator determined by IPA (Figure S15); score plot showing significant difference in metabolites (Figure S16); categorization of significant metabolites (VIP > 1) found in the ovary of the BPA lineage (Figure S17); the enrichment of metabolites is associated with several metabolic pathways (Figure S18); gene metabolite interaction network in the ovary of BPA lineage showing positive association of metabolites with gene expression (Figure S19); and mapping of differential metabolites and genes found in the carbon metabolism of cancer (Figure S20) (PDF)
