# Supplementary material for: The PCOS–NAFLD Multidisease Phenotype Occurred in Medaka Fish Four Generations after the Removal of Bisphenol A Exposure
Source: Environ Sci Technol. 2023 Aug 15;57(34):12602–19. doi: 10.1021/acs.est.3c01922 (PMC10469501; doi:10.1021/acs.est.3c01922)
Supplement: Supplementary file 1 — es3c01922_si_001.pdf [file es3c01922_si_001.pdf]

<u>**Supplementary Information**</u>

The PCOS-NAFLD multi-disease phenotype occurred in medaka fish four generations after the removal of bisphenol A exposure

Sourav Chakraborty, Santosh Anand, Beh Reh, Seraiah Coe, Ramji K. Bhandari‡

Department of Biology, University of North Carolina Greensboro, Greensboro, NC 27412, USA

‡ Corresponding Author: Ramji K. Bhandari (Email: rkbhanda@uncg.edu)

Number of pages: 27

Number of Figure: 31

Number of tables:1

Description of supporting information

Information provided in the supporting material section shows mechanisms associated with PCOS in the ovary of medaka fish whose ancestors were exposed to BPA four generations ago. The analysis was performed using the Qiagen IPA software, KEGG pathway analysis (open-source software), GSEA and metaboanalyst 5.0 software. Results are provided as disease network and pathways suggested by transcriptional and metabolomic analysis.

Contents

Table

1. Primers used in quantitative real-time PCR.

Figures

Figure S1. A global alteration in gene expression in the ovary of the BPA lineage. Heatmap showing the statistically significant differentially expressed genes (DEGs) in three biological replicates of the control and BPA-exposed lineages in the F4 generation.

Figure S2. Gene Ontology analysis in the ovary of BPA lineage. A. Cellular component, B. Molecular function, C. Biological pathway.

Figure S3. DEGs enriched in Insulin signaling pathway in the ovary of BPA lineage.

Figure S4. DEGs found in MAPK signaling pathway in ovary of BPA lineage.

Figure S5. DEGs enriched in AMPK signaling pathway in ovary of BPA lineage.

Figure S6. DEGs found in Rap1 signaling pathway in ovary of BPA lineage.

Figure S7. DEGs found in cAMP signaling pathway in ovary of BPA lineage.

Figure S8. Standard GSEA analysis and heatmap of enriched DEGs associated with pathogenesis in the ovary of BPA lineage. A) Autophagic mechanism B) cellular stress response C) Chromatin organization D) Cell signaling mechanism.

Figure S9. Standard GSEA analysis and heatmap of enriched DEGs. A) cell cycle B) catabolic process C) Plasma membrane component D) mitochondria metabolic pathway E) apoptosis F) p53 pathway

Figure S10. The PCOS-specific mutual DEGs in the ovary of the BPA lineage fish and PCOS patient dataset. A) Upregulated DEGs, B) Downregulated DEGs, C) BPA-specific downregulated DEGs, and D) BPA-specific upregulated DEGs.

Figure S11. Molecular mechanism of cancer triggering pathway in the ovary of the BPA lineage fish.

Figure S12. Molecular mechanism of autophagy triggering pathway in the ovary of the BPA lineage fish.

Figure S13. Molecular mechanism of HOTAIR mechanism in the ovary of the BPA lineage fish.

Figure S14. Gene disease network via ingenuity Pathway analysis (IPA) showing activation of akt1, tnf, and ifng associated with activation disease specific pathways.

Figure S15. Predicted top 15 upstream regulator determined by IPA. Upstream regulator mediated active target molecules and mechanistic network indicating potential involvement of upstream regulator in ovarian pathogenesis triggered by ancestral BPA exposure effect.

Figure S16. Score plot showing significant difference in metabolites in biological replicates of BPA lineage and control lineage fish.

Figure S17. Categorization of significant metabolites (VIP>1) found in the ovary of BPA lineage.

Figure S18. The enrichment of metabolites is associated with several metabolic pathways.

Figure S19. Gene metabolite interaction network in the ovary of BPA lineage showing positive association of metabolites with gene expression.

Figure S20. Mapping of differential metabolites (round blue circle) and genes (red triangle) found in carbon metabolism of cancer.

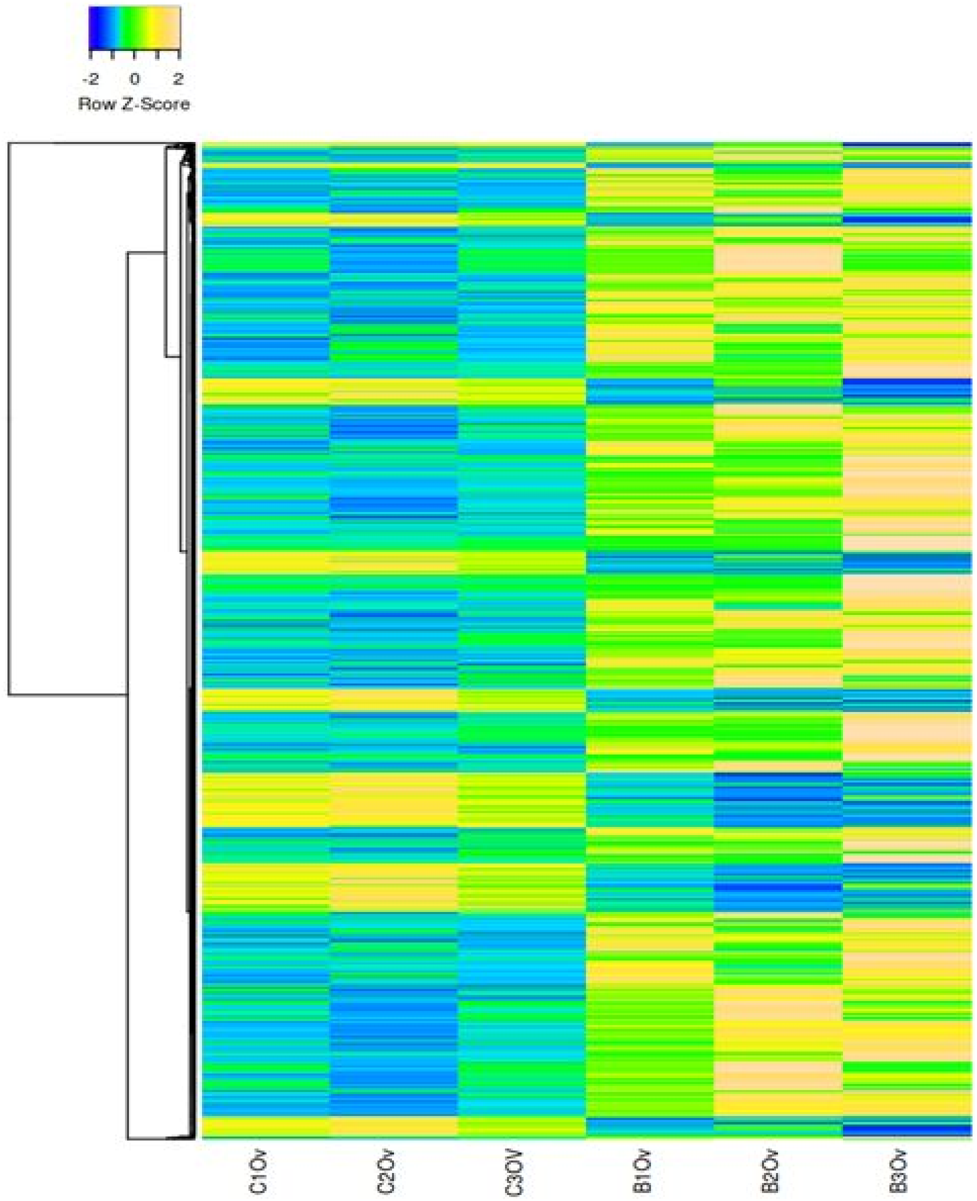

-2 0 2
Row Z-Score
C1Ov
C2Ov
C3OV
B1Ov
B2Ov
B3Ov

Figure S1. A global alteration in gene expression in the ovary of the BPA lineage. Heatmap showing the statistically significant differentially expressed genes (DEGs) in three biological replicates of the control and BPA-exposed lineages in the F4 generation. The RNA-seq analysis suggests an abundance of upregulated DEGs in the ovary of the BPA lineage fish compared to the control.

A

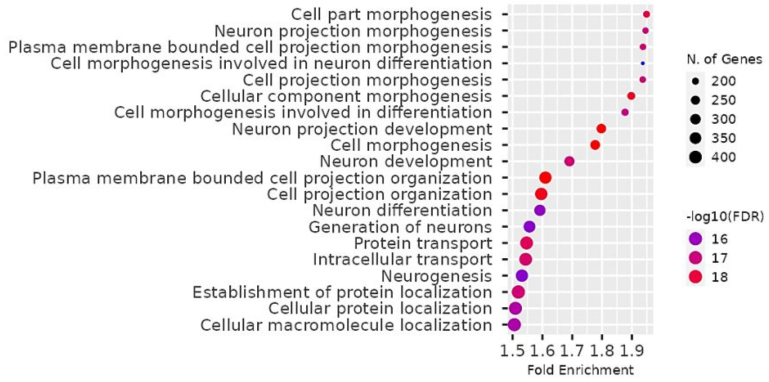

B

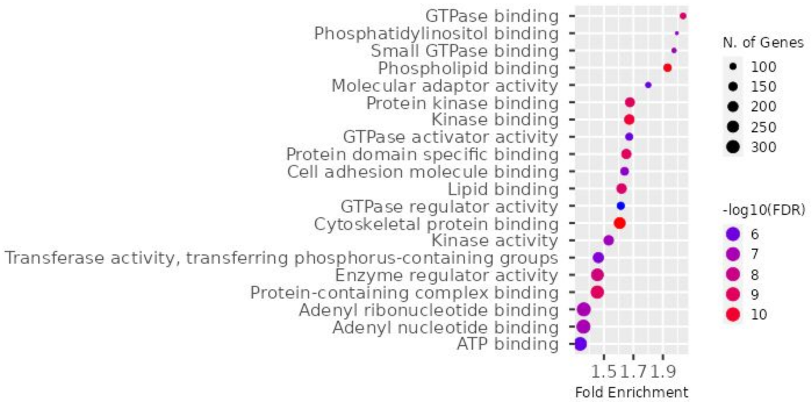

C

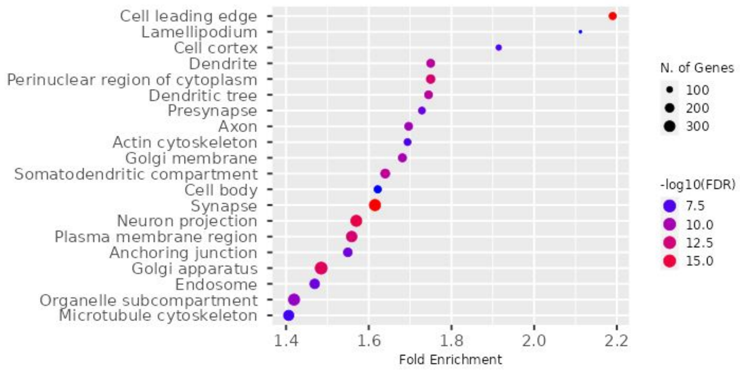

Figure S2. Gene Ontology analysis in the ovary of BPA lineage. A. Cellular component, B. Molecular function, C. Biological pathway.

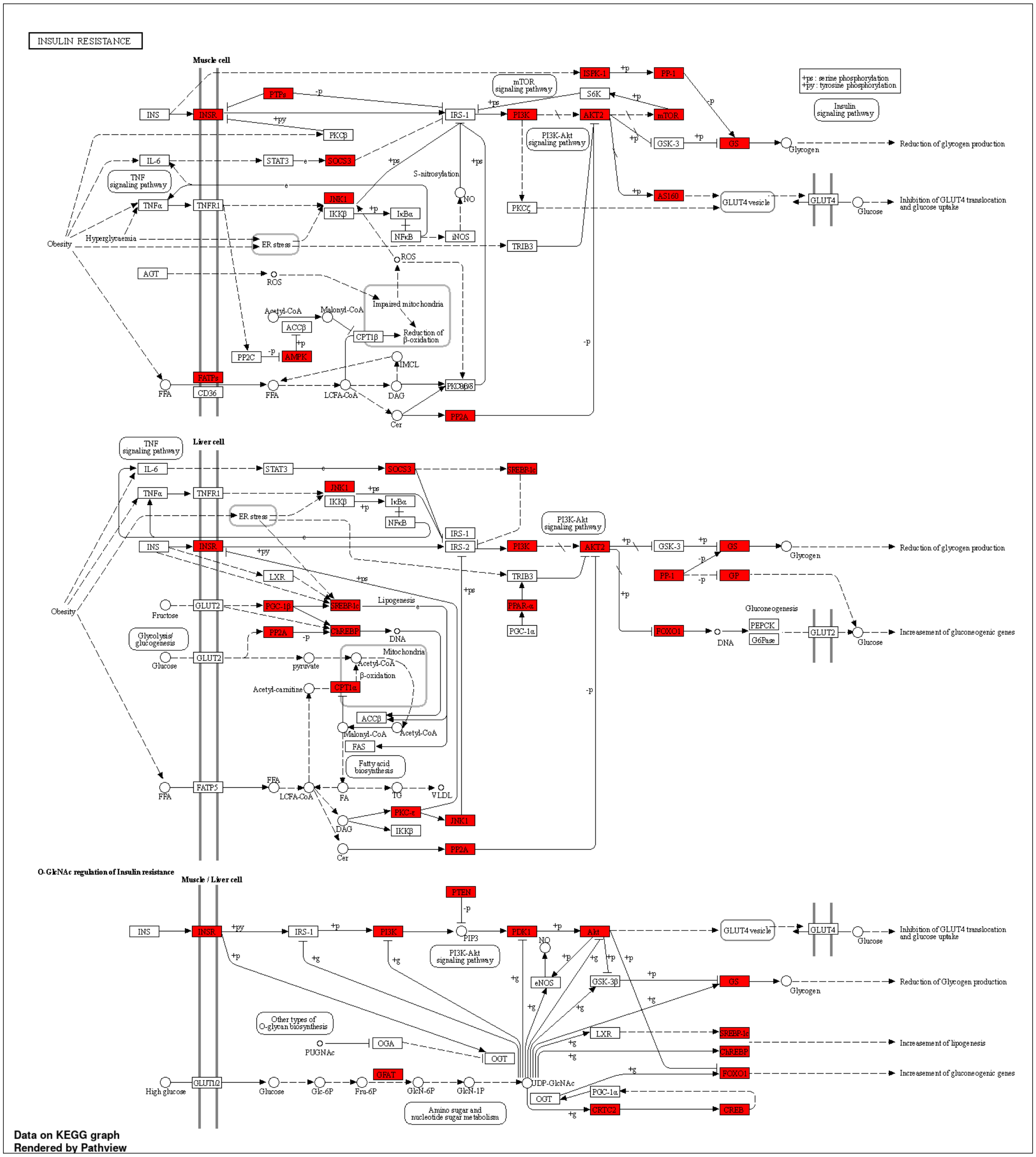

Figure S3. DEGs enriched in Insulin signaling pathway in the ovary of BPA lineage.

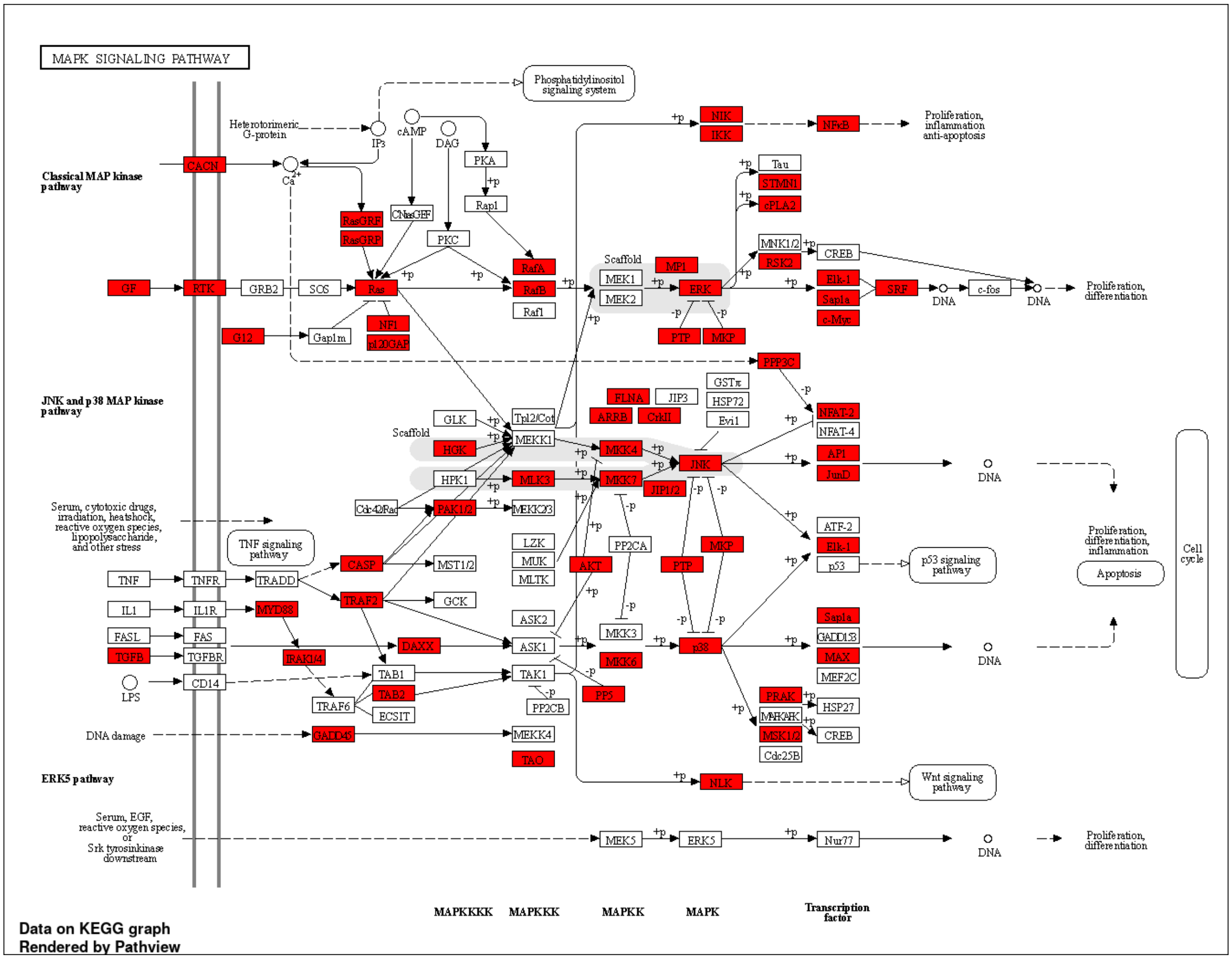

Figure S4. DEGs found in MAPK signaling pathway in ovary of BPA lineage.

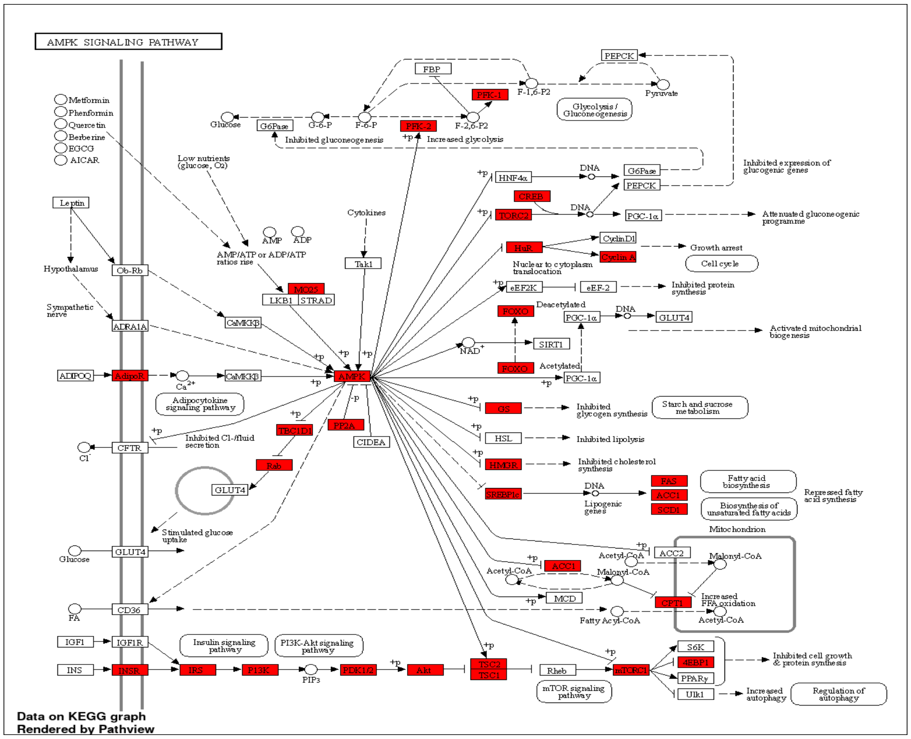

Figure S5. DEGs enriched in AMPK signaling pathway in ovary of BPA lineage.

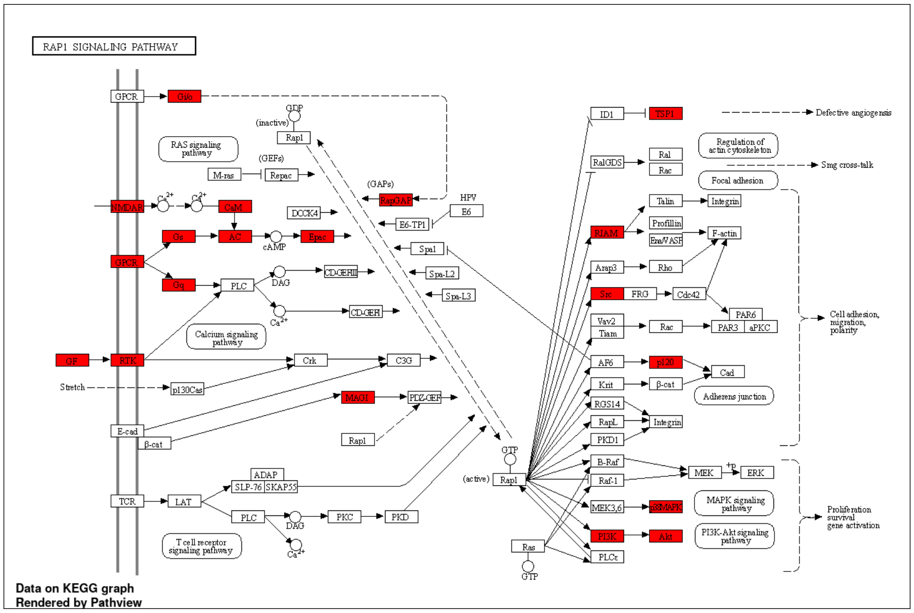

Figure S6. DEGs found in Rap1 signaling pathway in ovary of BPA lineage.

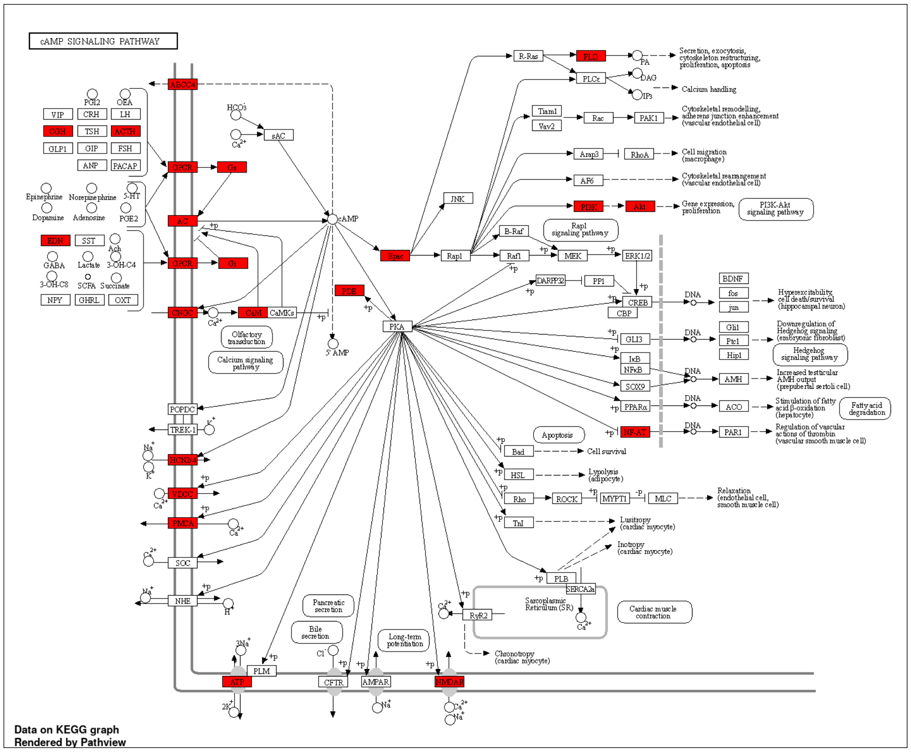

Figure S7. DEGs found in cAMP signaling pathway in ovary of BPA lineage.

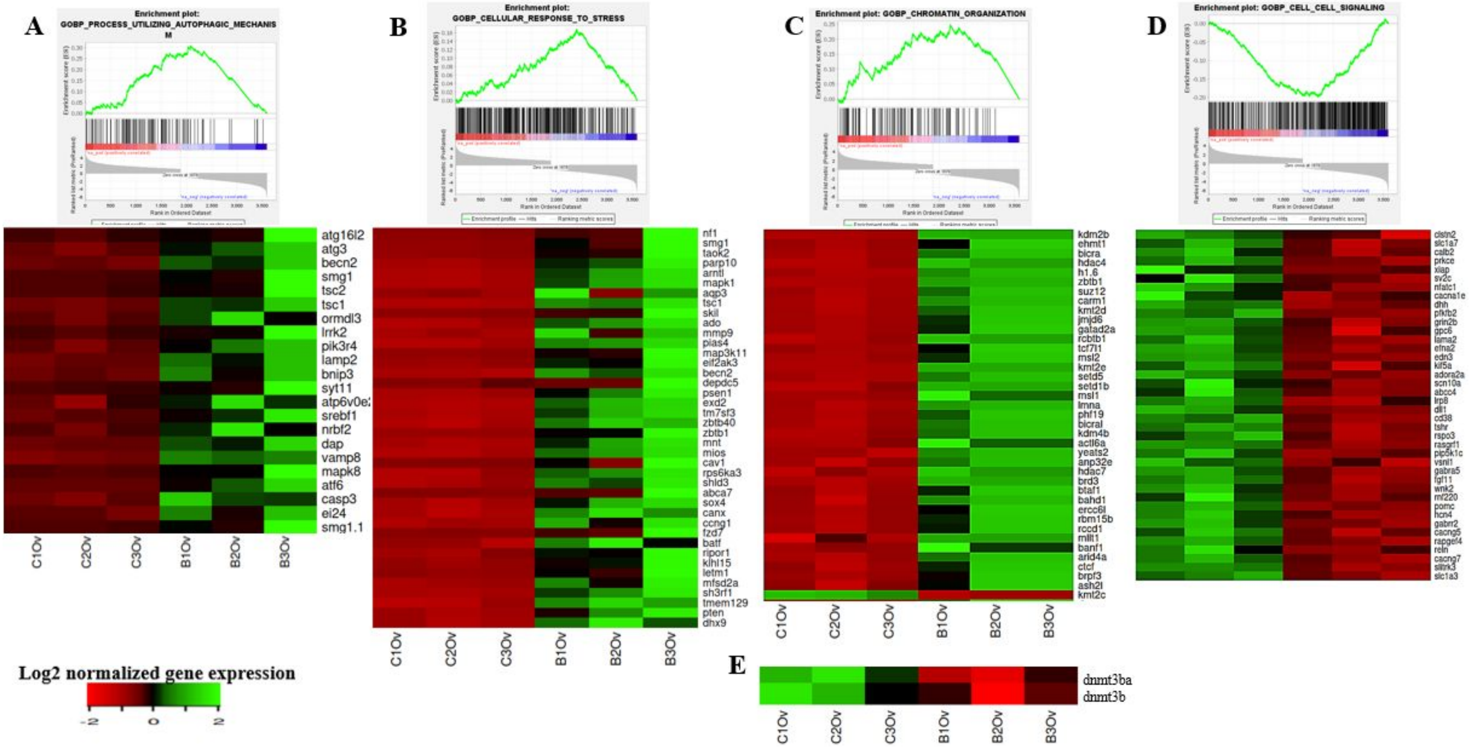

Figure S8. Standard GSEA analysis and heatmap of enriched DEGs associated with pathogenesis in the ovary of BPA lineage. A) Autophagic mechanism B) cellular stress response C) Chromatin organization D) Cell signaling mechanism.

A.

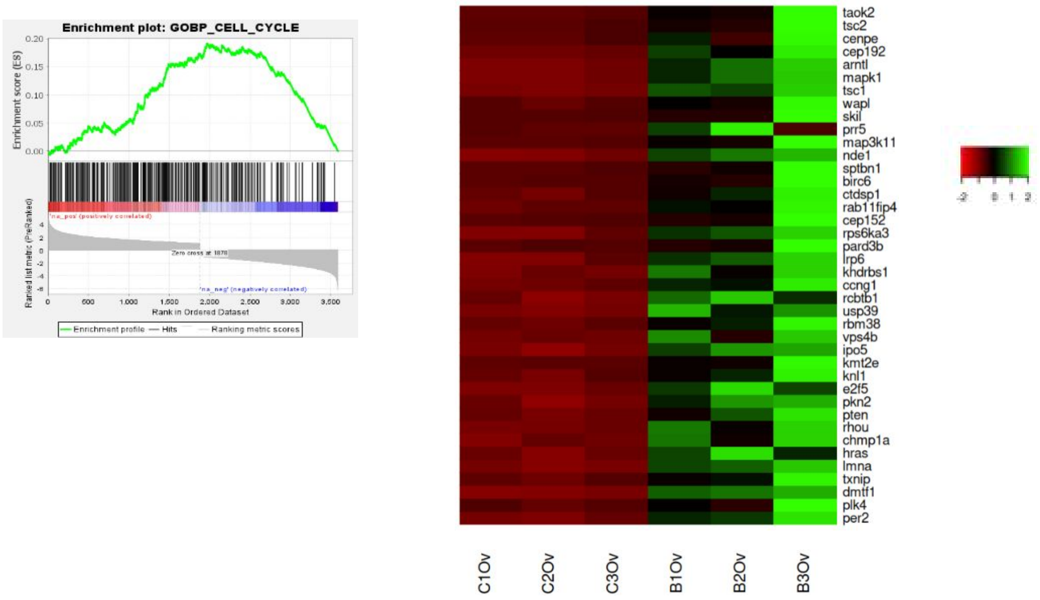
Enrichment plot: GOBP_CELL_CYCLE
Enrichment score (ES)
Ranked list metric (PreRanked)
'na_pos' (positively correlated)
Zero cross at 1878
'na_neg' (negatively correlated)
Rank in Ordered Dataset
Enrichment profile — Hits — Ranking metric scores
taok2
tsc2
cenpe
cep192
arntl
mapk1
tsc1
wapl
skil
prr5
map3k11
nde1
sptbn1
birc6
ctdsp1
rab11fip4
cep152
rps6ka3
pard3b
lrp6
khdrbs1
ccng1
rcbtb1
usp39
rbm38
vps4b
ipo5
kmt2e
knl1
e2f5
pkn2
pten
rhou
chmp1a
hras
lmna
txnip
dmtf1
plk4
per2
C1Ov
C2Ov
C3Ov
B1Ov
B2Ov
B3Ov

B.

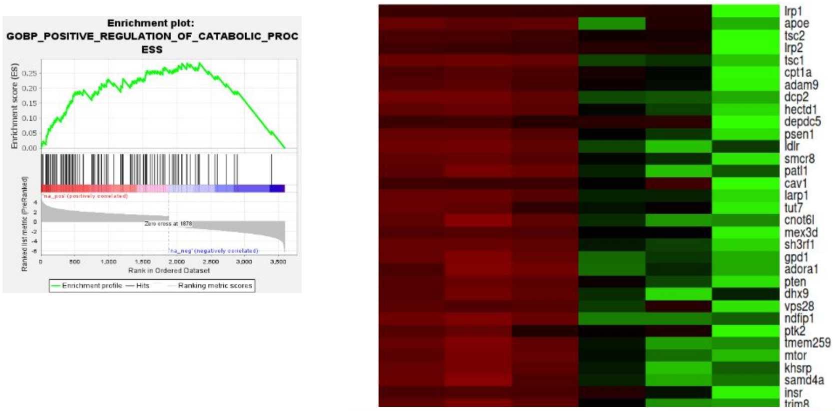
Enrichment plot:
GOBP_POSITIVE_REGULATION_OF_CATABOLIC_PROC
ESS
Enrichment score (ES)
Ranked list metric (PreRanked)
'na_pos' (positively correlated)
Zero cross at 1878
'na_neg' (negatively correlated)
Rank in Ordered Dataset
Enrichment profile — Hits — Ranking metric scores
lrp1
apoe
tsc2
lrp2
tsc1
cpt1a
adam9
dcp2
hectd1
depdc5
psen1
ldlr
smcr8
patl1
cav1
larp1
tut7
cnot6l
mex3d
sh3rf1
gpd1
adora1
pten
dhx9
vps28
ndfip1
ptk2
tmem259
mtor
khsrp
samd4a
insr
trim8

C.

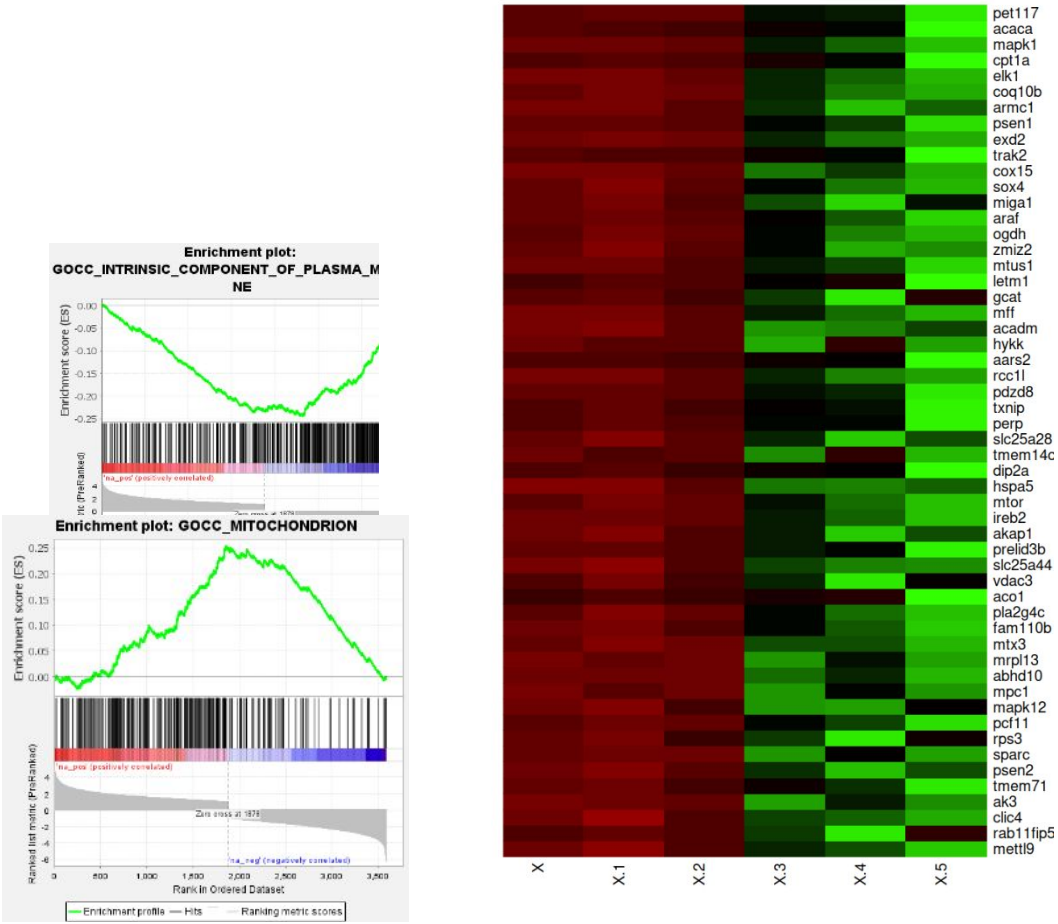
Enrichment plot:
GOCC_INTRINSIC_COMPONENT_OF_PLASMA_M
NE
Enrichment plot: GOCC_MITOCHONDRION
Enrichment score (ES)
Ranked list metric (PreRanked)
'na_pos' (positively correlated)
Zero cross at 1878
'na_neg' (negatively correlated)
Rank in Ordered Dataset
Enrichment profile — Hits — Ranking metric scores
pet117
acaca
mapk1
cpt1a
elk1
coq10b
armc1
psen1
exd2
trak2
cox15
sox4
miga1
araf
ogdh
zmiz2
mtus1
letm1
gcat
mff
acadm
hykk
aars2
rcc1l
pdzd8
txnip
perp
slc25a28
tmem14c
dip2a
hspa5
mtor
ireb2
akap1
prelid3b
slc25a44
vdac3
aco1
pla2g4c
fam110b
mtx3
mrpl13
abhd10
mpc1
mapk12
pcf11
rps3
sparc
psen2
tmem71
ak3
clic4
rab11fip5
mettl9
X
X.1
X.2
X.3
X.4
X.5

D.

C1Ov C2Ov C3Ov B1Ov B2Ov B3Ov

E.

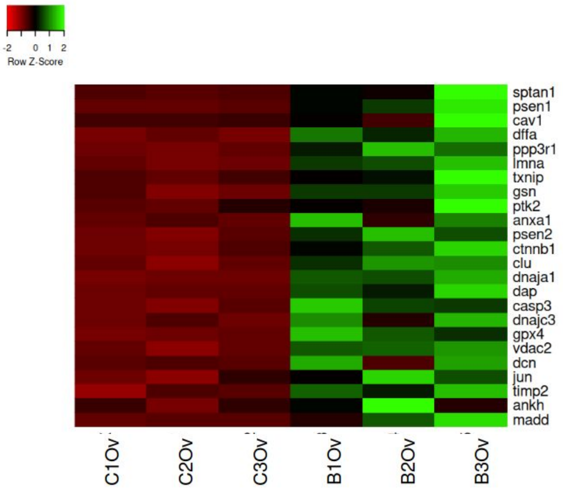

F.

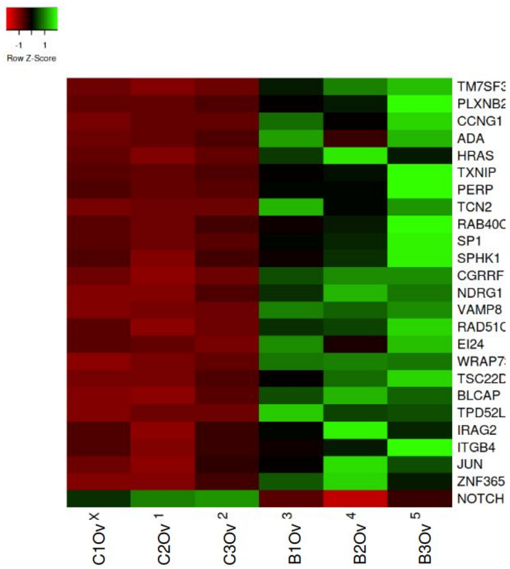

Figure S9. Standard GSEA analysis and heatmap of enriched DEGs. A) cell cycle B) catabolic process C) Plasma membrane component D) mitochondria metabolic pathway E) apoptosis F) p53 pathway

A.

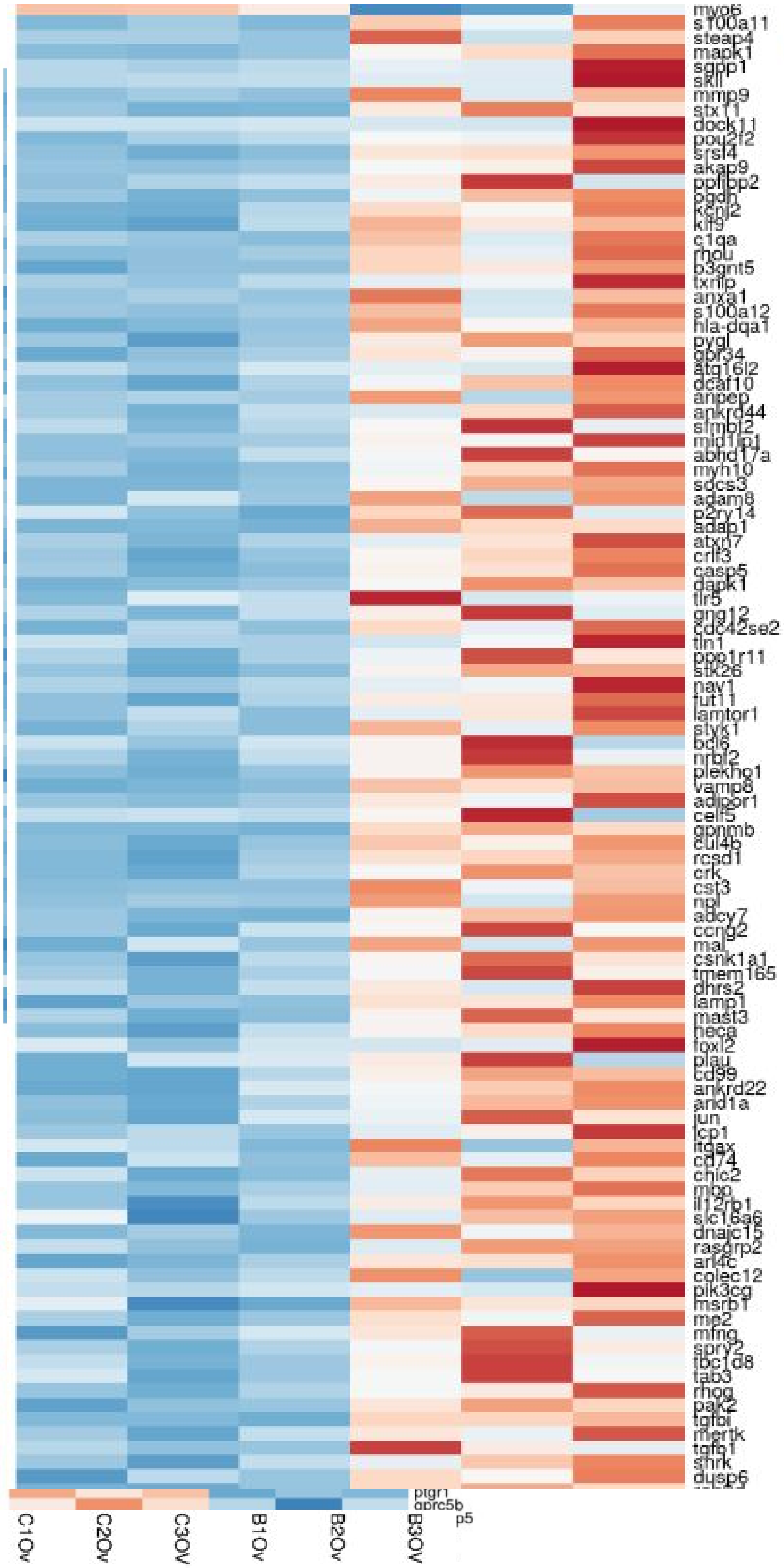
myo6
s100a11
steap4
mapk1
sgpp1
skil
mmp9
stx11
dock11
pou2f2
srsf4
akap9
ppfibp2
kcnj2
klf9
c1qa
rhou
b3gnt5
txnip
anxa1
s100a12
hla-dqa1
pygl
gpr34
atg16l2
dcaf10
anpep
ankrd44
mid1ip1
abhd17a
myh10
socs3
adam8
p2ry14
adap1
atxn7
crlf3
casp5
dapk1
tlr5
gng12
cdc42se2
tln1
ppp1r11
stk26
nav1
fut11
lamtor1
styk1
bcl6
nrbf2
plekho1
vamp8
adipor1
celf5
gpnmb
cul4b
rcsd1
crk
cst3
npl
adcy7
ccng2
mal
csnk1a1
tmem165
dhrs2
lamp1
mast3
heca
foxl2
plau
cd99
ankrd22
arid1a
jun
lcp1
itgax
cd74
chic2
mbp
il12rb1
slc16a6
dnajc15
rasgrp2
arl4c
colec12
pik3cg
msrb1
me2
mfng
spry2
tbc1d8
tab3
rhog
pak2
tgfbi
mertk
tgfb1
dusp6
ptgr1
gprc5b
p5
C1Ov
C2Ov
C3OV
B1Ov
B2Ov
B3OV

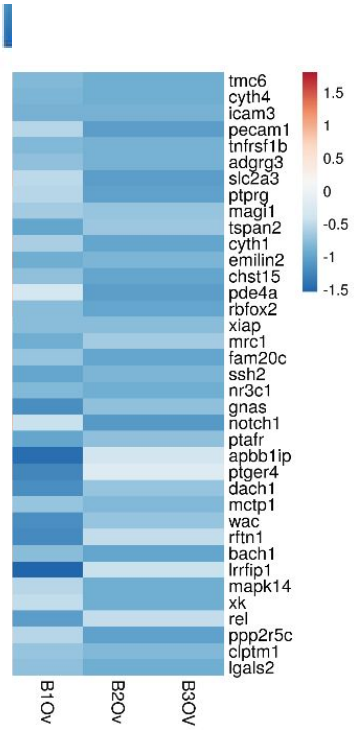
tmc6
cyth4
icam3
pecam1
tnfrsf1b
adgrg3
slc2a3
ptprg
magi1
tspan2
cyth1
emilin2
chst15
pde4a
rbfox2
xiap
mrc1
fam20c
ssh2
nr3c1
gnas
notch1
ptafr
apbb1ip
ptger4
dach1
mctp1
wac
rftn1
bach1
lrrfip1
mapk14
xk
rel
ppp2r5c
clptm1
lgals2
B1Ov
B2Ov
B3OV
1.5
1
0.5
0
-0.5
-1
-1.5

C.

C1Ov

C2Ov

C3OV

B1Ov

B2Ov

B3OV

Figure S10. The PCOS-specific mutual DEGs in the ovary of the BPA lineage fish and PCOS patient dataset. A) Upregulated DEGs, B) Downregulated DEGs, C) BPA-specific downregulated DEGs, and D) BPA-specific upregulated DEGs.

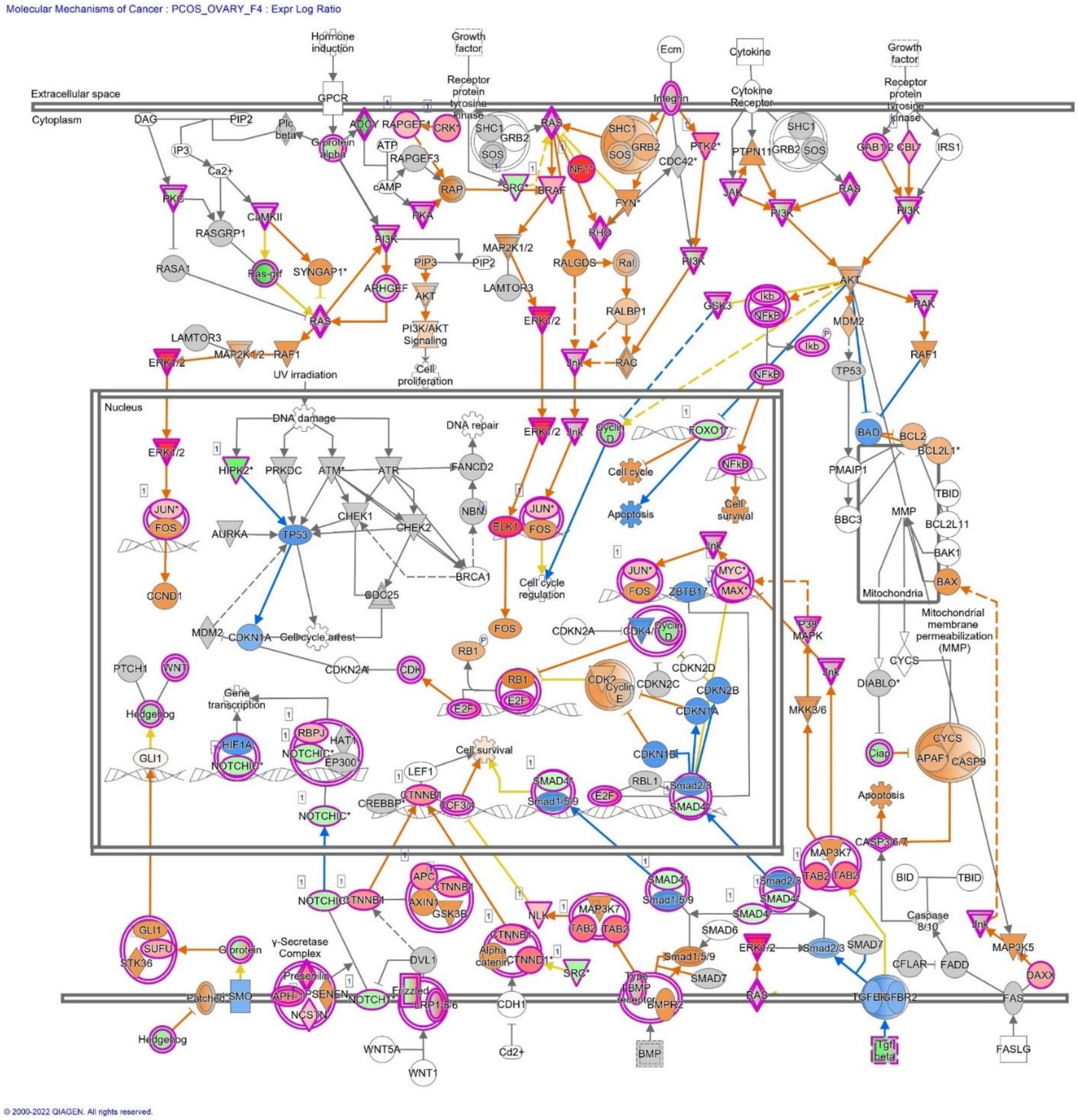

Figure S11. Molecular mechanism of cancer triggering pathway in the ovary of the BPA lineage fish.

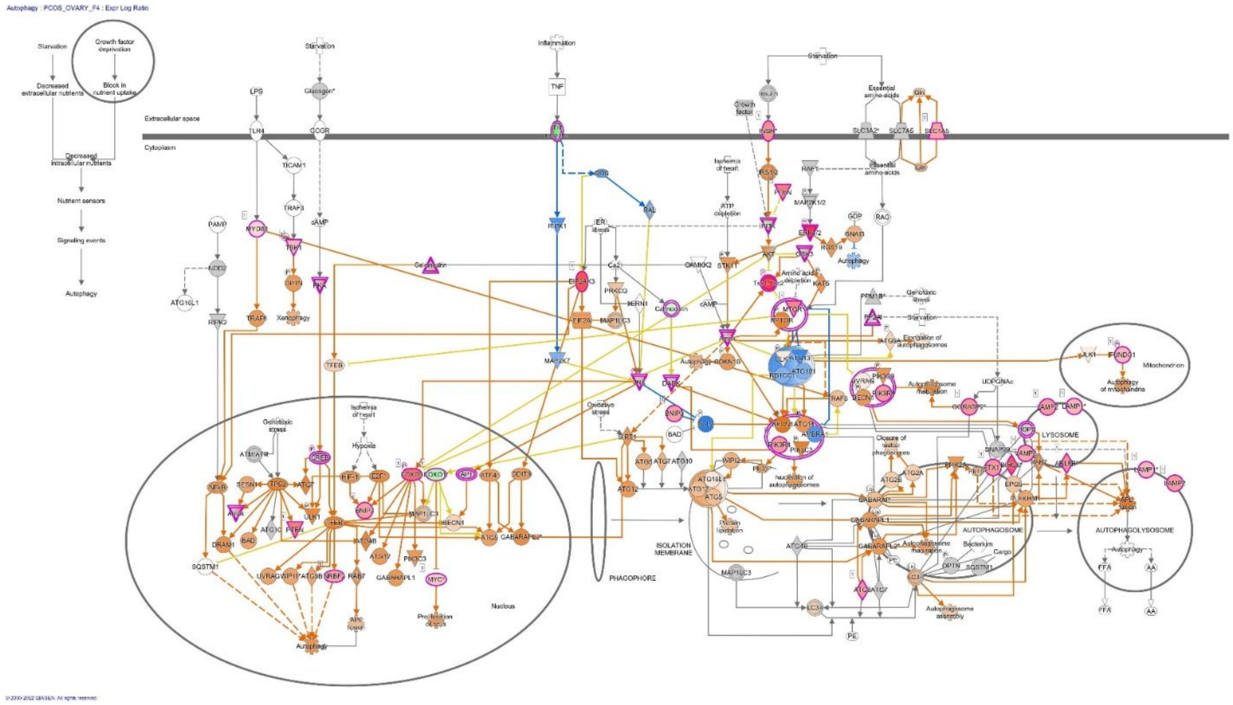

Figure S12. Molecular mechanism of autophagy triggering pathway in the ovary of the BPA lineage fish.

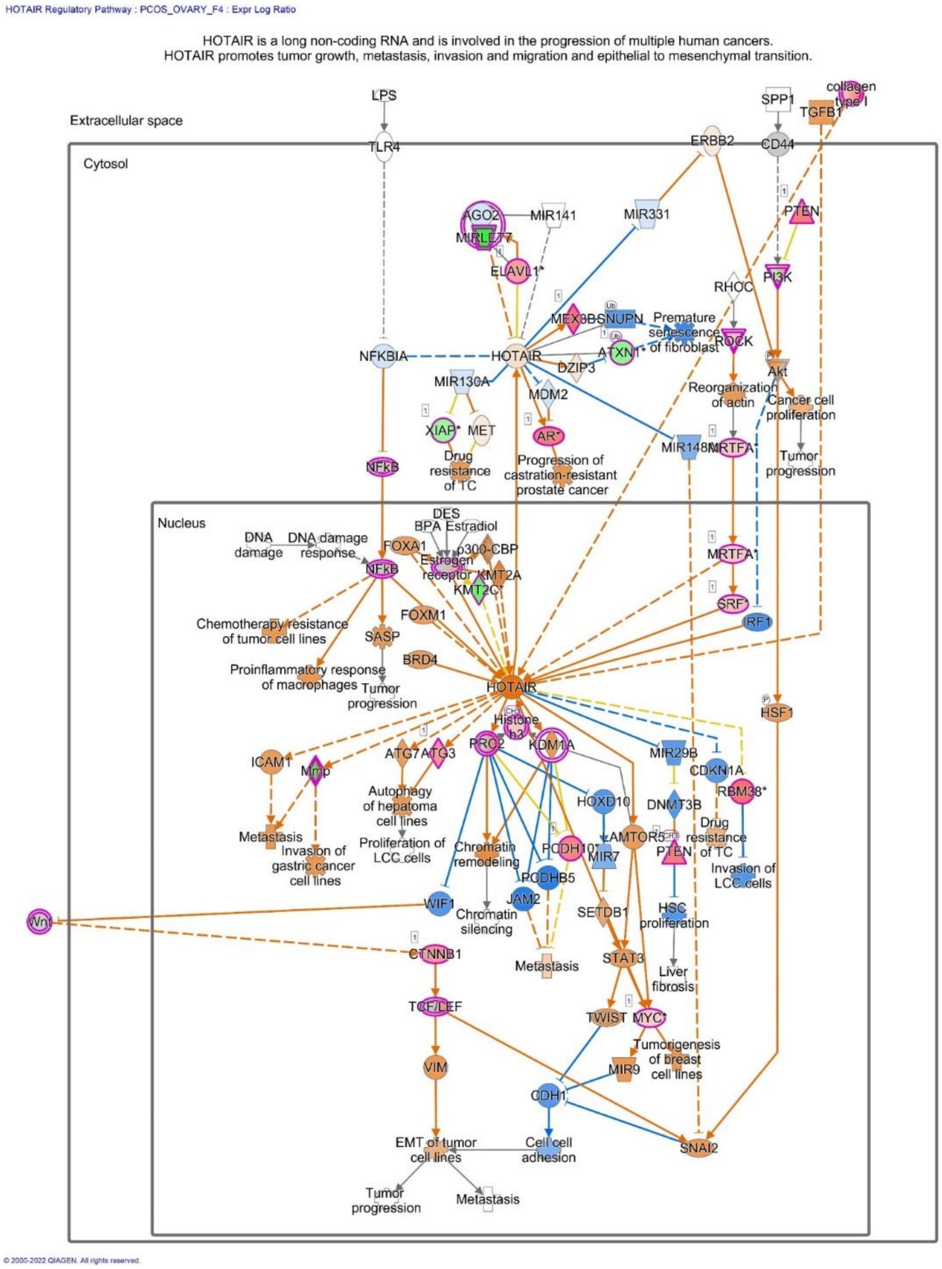

Figure S13. Molecular mechanism of HOTAIR mechanism in the ovary of the BPA lineage fish.

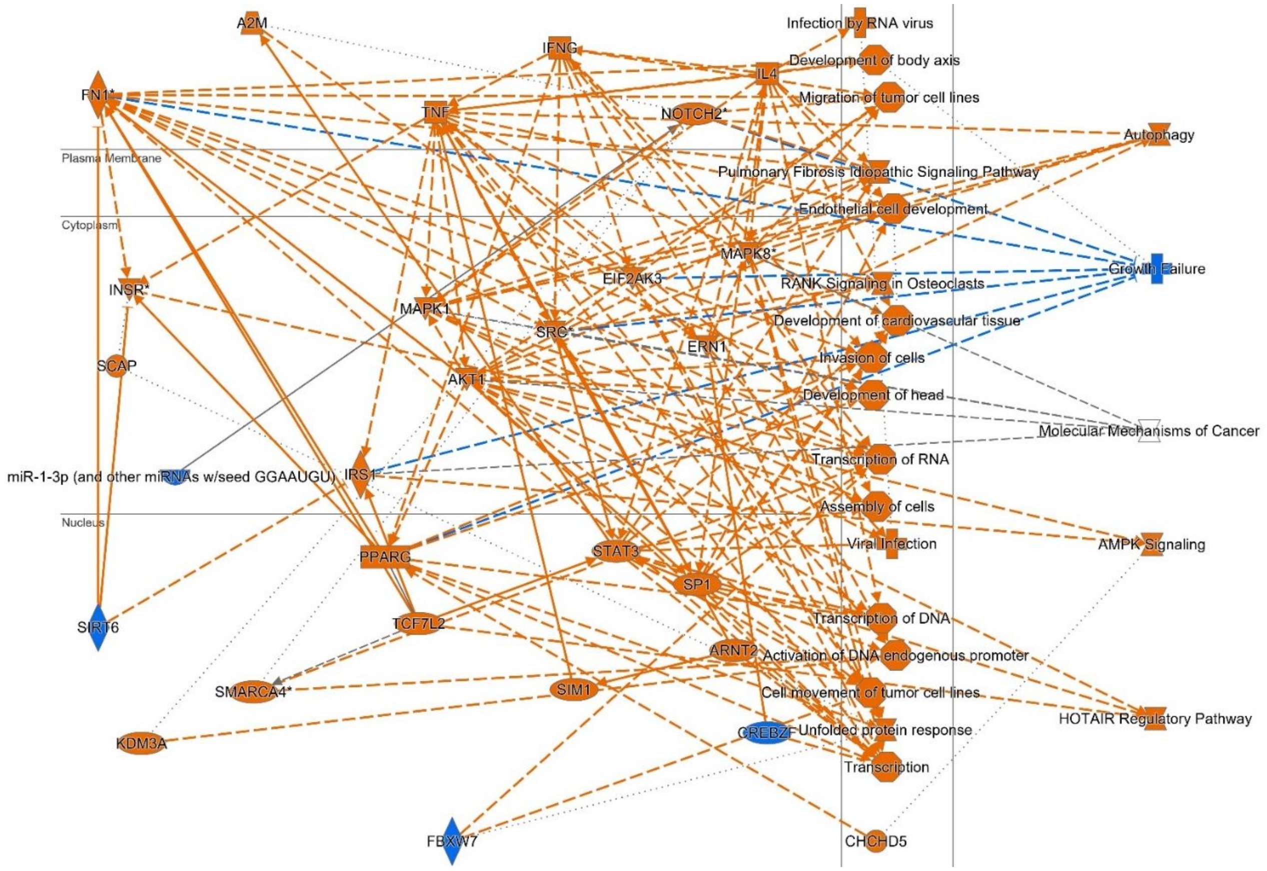

Figure S14. Gene disease network via ingenuity Pathway analysis (IPA) showing activation of akt1, tnf, and ifng associated with activation disease specific pathways.

Upstream Regulator	Expr Log Ratio	Molecule Type	Predicted	Activation	Flags	p-value of overlap	Target Molecules in Dataset	Mechanistic Network
ESR1	1.076	ligand-dependent nuclear r	Activated	2.877		4.4E-17	ABCA4,ABI2,ABLIM1,ACACA,ADA	1005 (17)
TGFB1	0.917	growth factor	Activated	3.843		6.33E-12	ABCA1,ABI2,ABLIM3,ACAA2,ACAC	1124 (21)
HNF4A	0.28	transcription regulator	Activated	2.216		1.22E-08	ABCB8,ABHD10,ACAA2,ACIN1,AC	1071 (19)
NPC1	-0.034	transporter	Inhibited	-3.543	bias	1.29E-08	ABCA1,ACKR4,APOB,APOE,ARNTL	555 (14)
HTT	1.302	transcription regulator	Activated	2.379		0.000000143	ABCA1,ACADM,ADORA2A,AGRN,A	869 (19)
EGFR	0.294	kinase	Activated	2.683	bias	0.000000364	ABI1,ACO1,AMOTL2,ANXA1,ANXA	779 (18)
HMG20A	0.871	transcription regulator	Activated	2.117	bias	0.0000123	ACSS2,ALDOC,CD9,CLU,DCN,ECM	
EIF2AK3	2.927	kinase	Activated	2.072	bias	0.0000189	ALDH18A1,ARNTL,ATF6,BET1L,CA	708 (17)
KLF3	-0.037	transcription regulator	Inhibited	-3.969		0.0000263	AP1M2,AP4M1,APOE,ARFGAP2,AR	
TSC2	3.874	other	Inhibited	-4.399	bias	0.000031	ACACA,ACSS2,ANXA1,ANXA2,AT	860 (20)
CST5	-0.53	other	Inhibited	-2.714	bias	0.0000386	ABCC1,ABLIM1,ANK3,ANXA2,AP1	
AR	2.197	ligand-dependent nuclear r	Activated	3.937	bias	0.0000455	ABCA1,ABCC4,ACTR3,AK3,ALOX1	928 (19)
SMAD3	0.346	transcription regulator	Activated	3.098	bias	0.0000508	ABI2,ACSS2,ADAM15,ADAM9,ADC	926 (18)
SORL1	0.33	transporter	Activated	3.212	bias	0.0000517	ABCA1,ACVRL1,ADAM9,APOE,C3,	752 (16)

Figure S15. Predicted top 15 upstream regulator determined by IPA. Upstream regulator mediated active target molecules and mechanistic network indicating potential involvement of upstream regulator in ovarian pathogenesis triggered by ancestral BPA exposure effect.

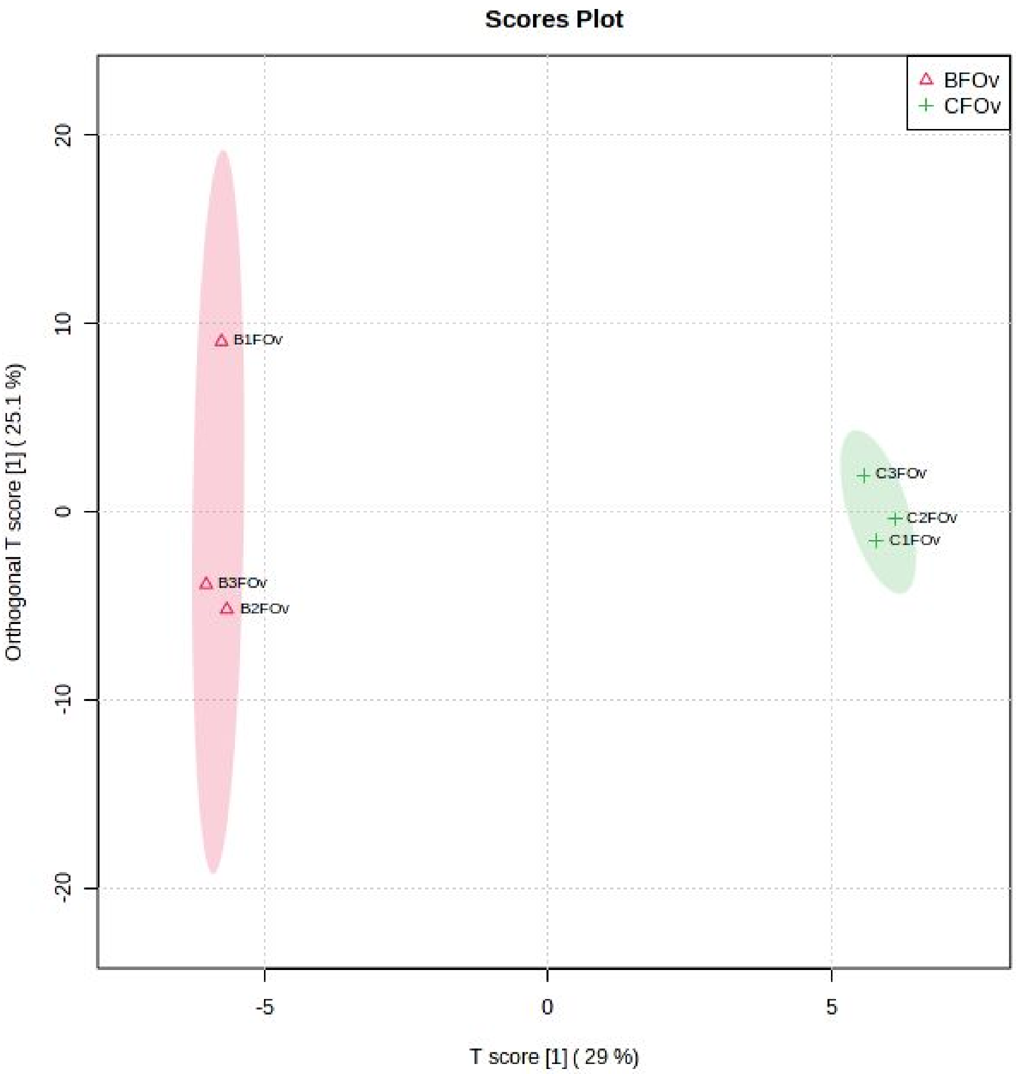

Figure S16. Score plot showing significant difference in metabolites in biological replicates of BPA lineage and control lineage fish.

HMDB-ID	Metabolites	VIP	m/z	Retention time	Class
HMDB0000158	Ornithine	1.7703	133.097	0.65	Amino acids, peptides, and analogues
HMDB0000791	Asymmetric dimethylarginine	1.70153	203.15	0.89	
HMDB0000379	Allysine	1.60517	146.081	6.18	
HMDB0000696	Phosphocreatine	1.5638	212.043	0.77	
HMDB0000133	L-Histidine	1.45792	156.077	1.14	
HMDB0000883	L valine	1.44683	118.087	0.89	
HMDB0002815	3-Dehydroquinate	1.42918	191.055	0.9	
HMDB0000001	1-Methylhistidine	1.41363	170.092	0.6	
HMDB0000157	N6-Acetyl-L-lysine	1.39034	189.123	0.75	
HMDB0000157	L-Aspartic acid	1.38007	134.045	0.67	
HMDB0000296	N2-Succinyl-L-ornithine	1.34437	233.113	0.89	
HMDB0001173	4-Guanidinobutanoic acid	1.28226	146.093	0.65	
HMDB0002820	Ne,Ne dimethyllysine	1.24632	175.144	1.71	
HMDB0000101	L-Glutamic acid	1.20576	148.06	0.67	
HMDB0000045	Creatine	1.07865	132.077	0.89	
HMDB0000132	L-Proline	1.05865	116.071	0.78	
HMDB0000251	Citrulline	1.01903	176.103	0.67	
HMDB0003464	S-(Formylmethyl)glutathione	1.01845	350.102	0.58	
HMDB0000562	N6,N6,N6-Trimethyl-L-lysine	1.00937	189.16	1.25	
HMDB0000195	Xanthine	1.74762	153.041	1.05	Purines and pyrimidines derivatives
HMDB0000094	Guanosine	1.72087	284.099	0.88	
HMDB0000157	Inosine	1.63124	269.088	0.89	
HMDB0000114	Hypoxanthine	1.49358	137.046	0.88	
HMDB0000205	Uridine	1.40514	245.077	0.7	
HMDB0000206	Uracil	1.32067	113.035	2.87	
HMDB0000288	Uridine 5'-monophosphate	1.29434	323.029	0.65	
HMDB0000212	Cytosine	1.2426	112.051	0.69	
HMDB0000195	Uridine diphosphate-N-acetylglucosamir	1.23055	608.089	0.65	
HMDB0000070	Deoxyadenosine	1.10575	252.109	0.89	
HMDB0000094	Guanine	1.06435	152.056	0.67	
HMDB0000064	Cytidine	1.02857	244.093	1.14	
HMDB0000684	Nicotinic acid	1.77411	124.04	1.25	organic acids and derivatives
HMDB0000630	Niacinamide	1.73124	123.056	0.65	
HMDB0000238	Taurodeoxycholic acid	1.55108	500.305	0.9	
HMDB0000208	2-Methylcitric acid	1.54507	207.05	0.61	
HMDB0002815	3-Dehydroquinate	1.42918	191.055	0.9	
HMDB0001406	cis-2-Methylaconitate	1.39274	189.039	0.65	
HMDB0000159	Oxoadipic acid	1.32137	161.045	0.73	
HMDB0000252	L-Tryptophan	1.22815	205.097	0.89	
HMDB0004586	N-Acetylneuraminate	1.0437	310.113	0.9	
HMDB0000034	Taurocholic acid	1.02267	516.299	3.9	
HMDB0001514	2-Acetolactate	1.01862	133.05	1.22	
HMDB0000034	Taurocholic acid	1.02267	516.299	3.9	
HMDB0001406	cis-2-Methylaconitate	1.39274	189.039	0.65	
HMDB0000238	Taurodeoxycholic acid	1.55108	500.305	0.9	
HMDB0000253	D-1-Piperideine-2-carboxylic acid	1.37791	128.071	0.88	
HMDB0000208	2-Methylcitric acid	1.54507	207.05	0.61	
HMDB0000099	Glucosylceramide	1.76298	644.511	0.74	Lipid and lipid like molecules
HMDB0000158	L-Palmitoylcarnitine	1.68108	400.342	0.93	
HMDB0000614	PS(16:0/16:0)	1.37437	834.529	10.078	
HMDB0002815	5-Acetamidovalerate	1.35002	160.097	1.87	
HMDB0000089	Glycerol 3-phosphate	1.32379	173.021	0.87	
HMDB0000076	Glycerylphosphorylethanolamine	1.22135	216.063	0.64	
HMDB0002183	6-(alpha-D-Glucosaminyl)-1D-myo-inos	1.73297	342.14	0.67	Carbohydrates and carbohydrate conjugates
HMDB0000158	N-Acetylgalactosamine	1.51357	222.097	10.61	
HMDB0000089	Fructose 6-phosphate	1.09928	261.037	0.87	
HMDB0000177	Sphinganine	1.45101	302.305	4.08	Amines
HMDB0001301	Phytosphingosine	1.09994	318.3	3.91	
HMDB0000630	Retinal	1.3711	285.221	0.66	Retinoids
HMDB0000929	All-trans-retinoic acid	1.62391	301.216	0.89	
HMDB0001257	4,6-Dihydroxyquinoline	1.57068	162.055	1.85	Quinolones and derivatives
HMDB0001138	Vitamin K1 2,3-epoxide	1.23599	467.351	0.89	Quinone and hydroquinone lipids
HMDB0001316	(R)-Salsolinol	1.19059	180.102	0.93	Tetrahydroisoquinolines
HMDB0000222	L-Kynurenine	1.05836	209.092	0.76	Carbonyl compounds
HMDB0001316	(R)-Salsolinol	1.19059	180.102	0.93	Tetrahydroisoquinolines

Figure S17. Categorization of significant metabolites (VIP>1) found in the ovary of BPA lineage.

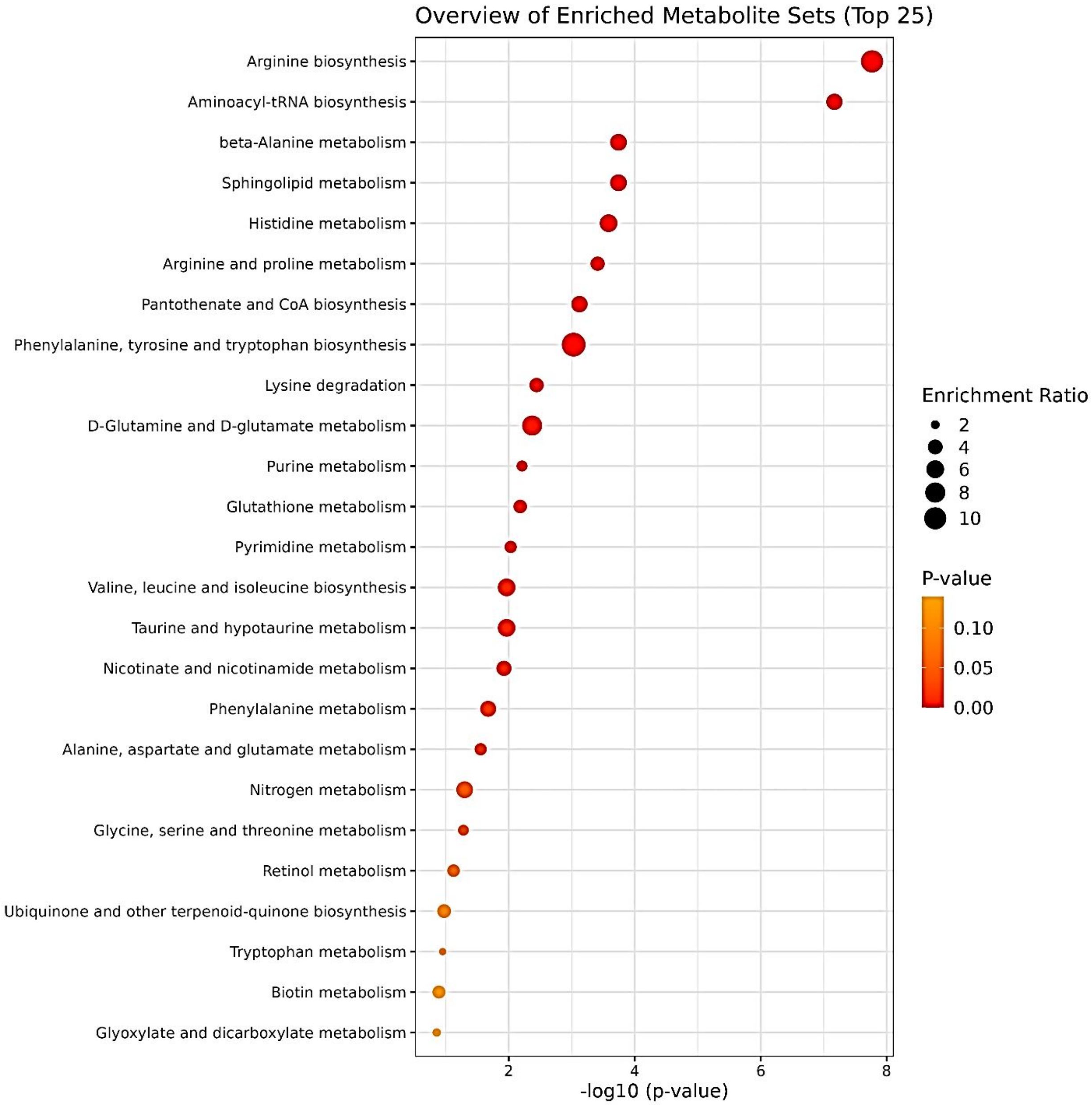

Figure S18. The enrichment of metabolites is associated with several metabolic pathways.

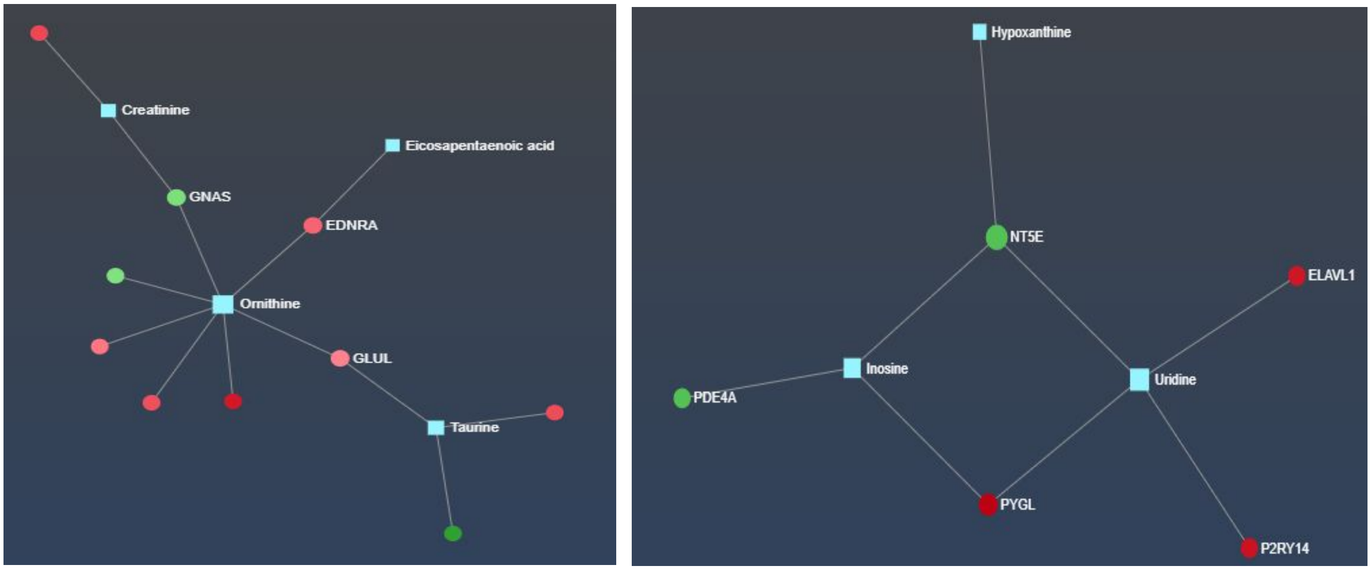

Figure S19. Gene metabolite interaction network in the ovary of BPA lineage showing positive association of metabolites with gene expression.

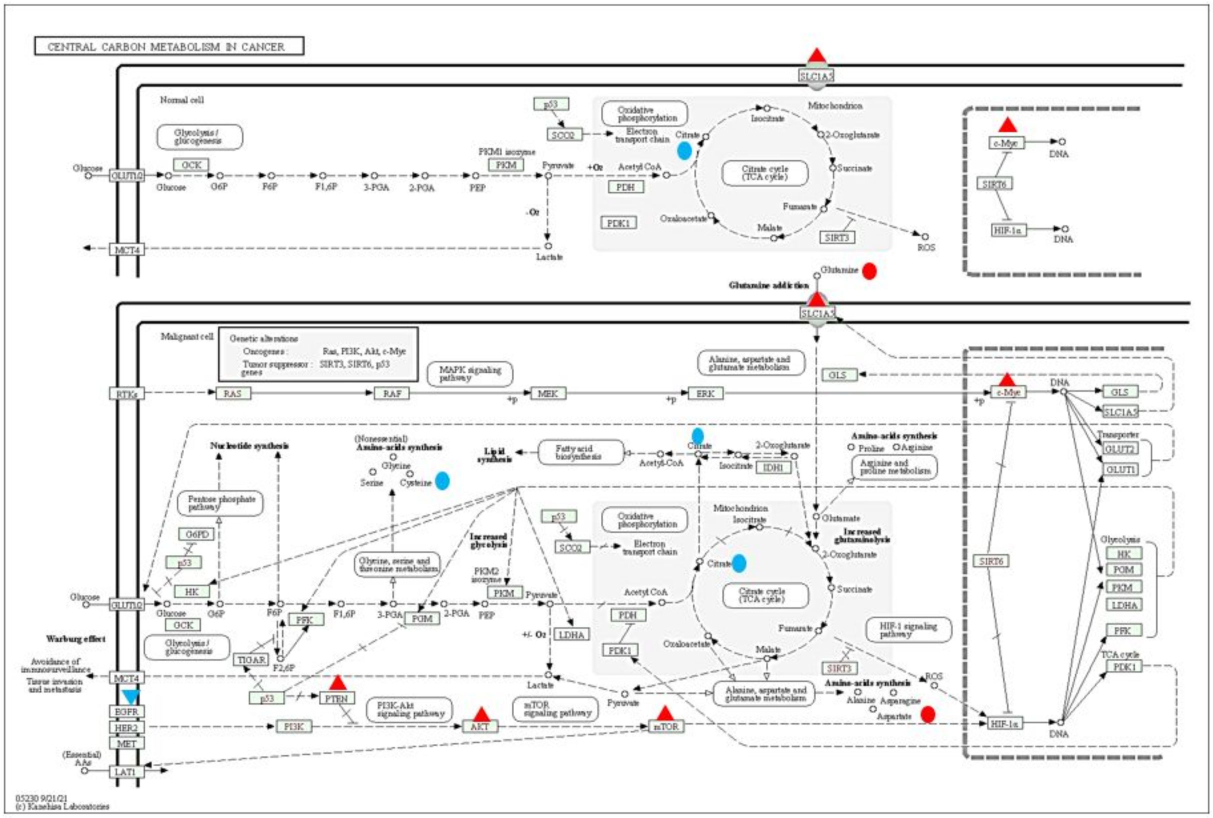

Figure S20. Mapping of differential metabolites (round blue circle) and genes (red triangle) found in carbon metabolism of cancer.

Table S1. Primers used in quantitative real-time PCR.

Gene name	Primer sequence 5`to 3`	Amplicon Size
lhr-f	TTTTGCCCTGAAAAGCCTCC	110
lhr-r	CTCTAAAACTTTGTTTCCGCCG	
3bhsd-f	AACAAACGTCTTCCACACCG	78
3bhsd-r	GCAGCTGCGTTCCTTTCAC	
fshr-f	TGAGTTGGTGGTGCTAGACA	53
fshr-r	CAACAGCTTCTTCAGGCCAC	
vldlr-f	TCGGTTCTGTGTGACTGACA	87
vldlr-r	ACTCCGTCTTTGTAGCGTGA	
casp3-f	GCTGTTTCGCCAAATGTCCA	105
casp3-r	CGATACCGGACCATCAGTGC	
shbg-f	TGATTGTTCAGTTTAAGCCGCA	113
shbg-r	AATCCTGTGCCCGAGAAGTA	
apoba-f	AAACCTGCATCATCCCATATCA	82
apoba-r	TGGTGATGTGACTTCCCTTG	
esr-1-f	AGCATCCAGGGTCACAATG	118
esr-1-r	AGCTCTTCCTCCGATTTCTGT	
vtg3-f	TTCCAAACAGAGAGGGGAGTC	123
vtg3-r	CCCTCAGTCGTCGGTTTGA	
18s rrna-f	CGTTCAGCCACACGAGATTG	56
18s rrna-r	CCGGACATCTAAGGGCATCA	
